# Chronic Alcohol Drinking Drives Sex-Specific Differences in Affective Behavior and Medial Prefrontal Cortex Activity in CRF1:Cre:tdTomato Transgenic Rats

**DOI:** 10.1523/ENEURO.0055-23.2023

**Published:** 2023-07-11

**Authors:** Sema G. Quadir, Gillian M. Arleth, Meredith G. Cone, Margaret W. High, Maria C. Ramage, Devin P. Effinger, Maria Echeveste Sanchez, Melissa A. Herman

**Affiliations:** 1 Bowles Center for Alcohol Studies, University of North Carolina Chapel Hill, Chapel Hill, NC 27599; 2 Department of Pharmacology, University of North Carolina Chapel Hill, Chapel Hill, NC 27599

**Keywords:** alcohol, anxiety, pain, corticotropin releasing factor receptor 1, prefrontal cortex, prelimbic, infralimbic

## Abstract

In 2021, 131 million adult Americans reported drinking alcohol in the last month, despite the well-known consequences of alcohol consumption. While alcohol use disorders (AUDs) are associated with both mood and chronic pain disorders, the relationship between alcohol drinking and affective and nociceptive behaviors remains unclear. Corticotropin releasing factor receptor-1 (CRF1) has been implicated in alcohol drinking, affective states, and pain sensitivity, often in a sex-dependent manner. In order to probe the effects of alcohol drinking on activity of CRF1+ cells and to also test the hypothesis that alcohol drinking is associated with both basal and subsequent affective and nociceptive readouts, we put male and female CRF1:cre:tdTomato rats through a battery of behavioral tests before and after intermittent access to alcohol. Following baseline testing, rats began alcohol (or water) drinking. Females consumed more alcohol in the first week, but there was no effect of sex on overall alcohol intake. Following three to four weeks of drinking, behavioral tests were repeated. Alcohol drinking decreased mechanical sensitivity, but no other effects of alcohol drinking were observed between experimental groups. Individual alcohol intake correlated with affective behavior in both sexes but only correlated with thermal sensitivity in males. There were no main effects of alcohol drinking or sex on CRF1+ neuronal activity in the medial prefrontal cortex (PFC) but final session alcohol intake correlated with activity in CRF1+ neurons in the infralimbic (IL) subregion. Together, our results suggest complex interplay between affective state, alcohol drinking, and the role of prefrontal CRF1+ neurons in mediating these behaviors.

## Significance Statement

Despite alcohol use being extremely comorbid with mood and pain disorders, there is still a limited understanding of the interaction and directionality between the them. To investigate this problem, rats were tested for affective behavior before and after being allowed to drink alcohol for six weeks. While baseline behavior did not predict subsequent intake, alcohol intake predicted subsequent affective behavior on an individual subject basis. These findings were accompanied by increased activity of the corticotropin releasing factor receptor-1 (CRF1) containing neurons in the infralimbic (IL) region of the prefrontal cortex (PFC). Together, these findings reveal a new mechanism for understanding alcohol use.

## Introduction

In 2021, 131 million adult Americans reported drinking alcohol (EtOH) in the last month, with almost 30 million adult Americans meeting the criteria for an alcohol use disorder (AUD; SAMHSA, 2022). While AUDs are clinically defined based on criteria such as inability to control drinking, tolerance, and withdrawal symptoms (American Psychiatric Association, 2013), they always begin with alcohol use that transitions into an AUD. Through repeated cycles of alcohol (EtOH) use, negative affective states can emerge on drinking cessation (Koob and Le Moal, 2005). These states can promote the development of dependence as individuals drink to alleviate withdrawal symptoms such as increased anxiety and anhedonia (Martinotti et al., 2008; Howe et al., 2021). Conversely, anxiety also often precedes EtOH use and is linked to a more rapid development and progression of AUD in both humans (Sloan et al., 2003; Sartor et al., 2007; Kushner et al., 2011; Boschloo et al., 2013) and rodents (Spanagel et al., 1995; Hayton et al., 2012; Pelloux et al., 2015). Mood disorders and EtOH use often occur together (Schellekens et al., 2015; Anker and Kushner, 2019; McHugh and Weiss, 2019; SAMHSA, 2022); however, our understanding of this relationship remains incomplete as both are influenced by a variety of other factors including sex. For example, genetic models demonstrated that rats bred for high anxiety-phenotypes or congenital learned helplessness drink more EtOH than rats bred for low-anxiety or noncongenital learned helplessness phenotypes, but both differences are only present in females (Vengeliene et al., 2005; Izídio and Ramos, 2007). Other studies found that affective behavior in the elevated plus maze predicts EtOH intake in male, but not female, rats (McNamara and Ito, 2021). In humans, EtOH use is more prevalent among men (SAMHSA, 2022); however, recent trends indicate that the prevalence of EtOH use among women is increasing at a faster rate than in men (Slade et al., 2016; Grant et al., 2017; Grucza et al., 2018) and the sex-related differences are narrowing (A.M. White et al., 2015; A. White, 2020; Keyes et al., 2021). Additionally, mood disorders are more common in women (Salk et al., 2017; Franceschini and Fattore, 2021) and women are more likely to drink heavily in response to psychological distress compared with their male counterparts (Olenick and Chalmers, 1991; Choi and DiNitto, 2011). Together, these studies highlight a major gap in our understanding of alcohol use and AUDs: how sex influences affective behaviors that both precede and follow EtOH use.

Affective behavior and EtOH use not only overlap in incidence, but also share similar neurobiological underpinnings. More specifically, both involve the stress-related corticotropin releasing factor (CRF) system. While hypothalamic release of CRF results in activation of the hypothalamic-pituitary-adrenal axis, extrahypothalamic CRF acts as an important neuromodulator in the brain through activity at two G-protein-coupled receptors, CRF1 and CRF2. In humans, polymorphisms in the gene encoding CRF1 are associated binge drinking, EtOH intake, and intoxication as well as with AUDs (Treutlein et al., 2006; A.C. Chen et al., 2010). Polymorphisms in CRF1 are also associated with affective behaviors such as post-traumatic stress disorder, panic disorder, depression, suicidality, and loneliness (Bradley et al., 2008; Keck et al., 2008; Wasserman et al., 2009; Grabe et al., 2010; Kranzler et al., 2011; Chou et al., 2014; Weber et al., 2016).

In rodents, systemically administered CRF1 antagonists decrease negative affect, EtOH intake, EtOH-induced negative affect and EtOH-induced hyperalgesia (Valdez et al., 2002; Breese et al., 2005; Overstreet et al., 2005; Sparta et al., 2008; Lowery et al., 2010; H. Wang et al., 2011; Edwards et al., 2012). CRF1 is found extensively throughout the brain and has been studied in the context of both EtOH drinking and affective behaviors. However, most studies focus only on the classical stress regions, such the bed nucleus of the stria terminalis (M.M. Huang et al., 2010; Faria et al., 2016; Rinker et al., 2017) and the amygdala (M.M. Huang et al., 2010; Koob, 2010; Kreifeldt et al., 2022; Rodriguez et al., 2022) despite CRF1 being abundantly expressed in cortical regions (Hubbard et al., 2011; Gozzi et al., 2013; Rieger et al., 2022). Indeed, alcohol preferring rats have increased CRF1 levels in the cingulate cortex, motor cortex, and somatosensory cortex (Hansson et al., 2006). CRF1 is also abundantly expressed in the medial prefrontal cortex (PFC; Brufatto et al., 2021; Weera et al., 2022); however, there are limited studies examining the effects of chronic EtOH drinking on CRF1-containing neurons in the PFC. The PFC’s role in mediating affective behavior (Klein et al., 2010; van Harmelen et al., 2010; Myers-Schulz and Koenigs, 2012; Carlson et al., 2015; Murrough et al., 2016; Nie et al., 2018; Wohleb et al., 2018; Naylor et al., 2019; Hare and Duman, 2020; Price and Duman, 2020; Jacobs and Moghaddam, 2021; Papasideris et al., 2021; Y. Chen et al., 2022; Dang et al., 2022; Dong et al., 2022; Kenwood et al., 2022; Yu et al., 2022; Bouras et al., 2023; R. Wang et al., 2023), nociception (Perlaki et al., 2015; S. Huang et al., 2019, 2020; D. Kang et al., 2019a, 2021; Ong et al., 2019; Tu et al., 2019; Kummer et al., 2020; C. Wang et al., 2020; Jefferson et al., 2021; Jiang et al., 2021; Malvestio et al., 2021; Mecca et al., 2021; Cao et al., 2022; Chang et al., 2022; Raisian et al., 2022; Ujcikova et al., 2022), and the effects of EtOH (Abernathy et al., 2010; Lu and Richardson, 2014; Tapocik et al., 2014; H. Zhang et al., 2014; Johnson et al., 2015; Linsenbardt and Lapish, 2015; Faccidomo et al., 2016; Natividad et al., 2018; Hughes et al., 2019, 2020, 2021; Scarlata et al., 2019; Barbier et al., 2021; Fish and Joffe, 2022; Zhao et al., 2022; Y. Hu et al., 2023) has been studied extensively, making this a prime region to investigate the relationship between these behaviors.

The present study uses behavioral testing and voluntary EtOH drinking in a CRF1 transgenic rat line to investigate the interaction between EtOH drinking and affective behavior in male and female rats. This study also examines the impact of chronic EtOH drinking on activity in the prefrontal cortex overall and specific to the CRF1 population. We specifically tested the hypothesis that basal affective and nociceptive behavior would predict subsequent drinking and that increased EtOH intake would further drive affective and nociceptive readouts. A period of three to four weeks of drinking was chosen as other papers found this time was sufficient to induce affective and nociceptive behaviors in both rats (Knapp et al., 2011; Gomez et al., 2013; Sharma et al., 2015; Thakore et al., 2016; Junqueira-Ayres et al., 2017) and mice (van Rijn et al., 2010; Lee et al., 2016, 2017; S. Liu et al., 2018; Szumlinski et al., 2019; Nennig et al., 2020; Bloodgood et al., 2021; N. Wang et al., 2021). Additionally, we chose to use the CRF1:cre:tdTomato rat to investigate the effects of voluntary EtOH drinking on activity in specific CRF1-containing neuronal population. Using this transgenic line also allows us to lay the groundwork for future studies involving manipulations of specific CRF1-containing populations and the effect on EtOH intake. We hypothesized that chronic EtOH drinking would increase activity of CRF1-containing neurons in the PFC in a dose-dependent manner (i.e., increased EtOH intake correlating with increased activity).

## Materials and Methods

### Subjects

A total of 32 adult male and female (*n* = 16/sex) CRF1:cre:tdTomato rats (Weera et al., 2022; 8–11 weeks old, bred in-house) were housed in temperature and humidity-controlled rooms with 12/12 h light/dark cycle (7 A.M. lights on). Rats had *ad libitum* access to food and water unless otherwise stated. Rats were handled before behavioral testing intake began. All animal procedures were performed in accordance with the University of North Carolina Chapel Hill animal care committee’s regulations.

### Splash test

Rats were allowed to habituate in the behavioral testing room for 1 h before testing. Rats were sprayed once with 10% w/v sucrose solution (∼5-cm spray distance, ∼100 μl per spray) on the dorsal coat of the torso, placed into a Plexiglas/textured flooring behavioral testing chamber [50 cm (length) × 50 cm (width) × 38 cm (height)] and recorded for 10 min in red light conditions. Latency to groom and total time spent grooming were scored by an experimenter blind to experimental group using the Behavioral Observation Research Interactive Software (BORIS) program (Friard and Gamba, 2016). Latency to groom and time spent grooming were used as measures of affective behavior.(Isingrini et al., 2010; Rosa et al., 2014; Engel et al., 2016; Haj-Mirzaian et al., 2016; Sadeghi et al., 2016; C. Hu et al., 2017; Lieberknecht et al., 2017; Andalib et al., 2019; Sasibhushana et al., 2019; Bijani et al., 2022; Quadir et al., 2022) One subject was excluded from analysis because of equipment malfunction during testing.

### Mechanical sensitivity testing (Von Frey)

Rats were brought into behavioral testing room 1 h before testing and were habituated for 15 min to a stainless-steel table (78 × 32 × 19.5 cm) with a perforated sheet containing staggered holes (0.28 cm diameter). Plastic filaments of increasing force (Bioseb EB2-VFF) were applied perpendicularly to hind paws until bending, and nocifensive responses defined as shaking, licking, or withdrawing the paw were recorded. Each paw was prodded three times/filament, beginning with the 2-g filament and increasing until the rat exhibited a nocifensive response during at least two trials. Withdrawal thresholds for each hind paw were then averaged for each animal to determine the paw withdrawal threshold.

### Novelty suppressed feeding (NSF)

Rats were given 1 Froot Loop in the home-cage 48 h before testing to prevent neophobia. Twenty-four hours before testing, the food was removed from the cage. On test day, rats were habituated to behavioral testing room for 1 h before testing. During testing, rats were individually placed into a brightly lit (150 lux) behavioral testing chamber (50 × 76 × 40 cm) containing one Froot Loop on filter paper in the center. Latency to eat was recorded and used as a measure of affective behavior (David et al., 2009; Iijima et al., 2012; J. Liu et al., 2015; Brachman et al., 2016; Barbieri et al., 2021; Zhou et al., 2021). After feeding initiation (or after 10 min), rats were removed from the chamber and returned to the home-cage where post-test consumption of preweighed Froot loops was recorded for 10 min. Post-test consumption was used as a measure of appetite (Iijima et al., 2012; P. Zhang et al., 2018).

### Thermal sensitivity testing (Hargreaves test)

Rats were assessed for thermal sensitivity using the Plantar Analgesia Meter (IITC). Rats were brought into the testing room 1 h before testing and habituated to the heated glass (temperature: 32°C) apparatus for 20 min. Infrared light (artificial intensity: 40) was focused onto each hindpaw and withdrawal latency was recorded. A maximum cutoff of 20 s was used to prevent tissue damage. Each hindpaw was tested twice. If the latencies differed by more than 1 s, then the paw was tested one additional time. Withdrawal latencies were averaged per paw then per animal.

### Intermittent access to two-bottle choice (IA2BC)

After 4 d of acclimatization to drinking out of two water bottles, one bottle containing 20% EtOH and one bottle of water were placed on cages for 24 h. This occurred on alternating days (MWF) and bottles were introduced or removed at 10 A.M., 3 h after onset of the light cycle [zeitgeber time (ZT) 3]. Water drinking rats had their water bottles changed every 24 h (at the same time) to ensure comparable treatment. Bottle placement was alternated to eliminate side preference; 20% v/v EtOH was prepared by diluting 95% EtOH (Pharmco Products Inc) in tap water.

### Behavioral testing following IA2BC

Behavioral testing began 24 h after EtOH bottles were removed and lasted 2–3 h. IA2BC resumed the following day. The order of testing was randomized at baseline. During postconsumption testing, pain tests occurred before affective testing to prolong the time between re-exposure to anxiogenic contexts. Finally, the NSF test was performed last as food deprivation is a known stressor (Genn et al., 2003; Nowland et al., 2011; Lenglos et al., 2013; Prvulovic et al., 2022) and could have confounded the other behavioral tests.

### Immunohistochemistry

Twenty-four hours after the last alcohol presentation, rats were anesthetized with isoflurane and transcardially perfused using PBS followed by 4% paraformaldehyde. Brains were postfixed in 4% PFA at 4°C overnight and then transferred to 30% sucrose in PBS at 4°C until brains sank. Brains were serially sectioned at 40 μm and stored in cryoprotectant (30% sucrose, 30% ethylene glycol, 0.01% sodium azide in PBS) at 4°C.

For each animal, four to six sections containing the medial prefrontal cortex (ranging from 3.72 to 2.52 mm anterior of bregma based on Paxinos and Watson, 2007) were used. These 40-μm sections underwent PBS washes and were incubated in 50% methanol for 30 min. Slices were blocked in 3% hydrogen peroxide for 10 min then 3% normal goat serum, 1% bovine serum albumin, 0.3% Triton X-100 in PBS for 1 h (with PBS washes in-between). Slices were incubated at 4°C for 48 h with rabbit anti-cFos primary antibody (1:3000, Millipore Sigma ABE457) and mouse anti-RFP primary antibody (1:500, Invitrogen MA5-15257) in blocking solution. Slices were then washed with 0.1% Tween 20 in Tris-buffered saline (TNT) before 30 min incubation in TNB blocking buffer (PerkinElmer FP1012). Next, slices were incubated with goat anti-rabbit horseradish peroxidase (1:400, Abcam ab6721) and goat anti-mouse 555 (1:800, Invitrogen A2123) in TNB buffer for 2 h before TNT washes. Lastly, slices were incubated in tyramide-conjugated fluorescein (Akoya Biosciences, NEL741001KT, 1:50) for 10 min. Following TNT washes, slices were mounted and sealed with mounting medium (Vector Laboratories H1500). Slides were stored at 4°C until imaging with the Keyence BZ-X800 fluorescence microscope.

Imaging and quantification were performed by an experimenter blind to the condition and sex of each animal. Images were taken at 20× and stitched together using the BZ-X800 Analyzer program. One hemisphere was selected at random for quantification, where the area of interest was outlined and counted manually using the FIJI multipoint counter tool (Schindelin et al., 2012). Cell counts were averaged per animal.

### Statistical analysis

All statistical analysis was performed using GraphPad Prism 8.0. In all studies, the threshold for significance was set to *p *<* *0.05. Data were assessed for normality using the Shapiro–Wilk test or D’Agostino and Pearson. For data that did not follow a normal distribution, nonparametric tests were used as described below. Detailed statistics for each experiment can be found in Table 1 and are referenced by superscripts in Results.

**Table 1:** Statistics table

Letter	Figure	Distribution [Shapiro–Wilk test or D’Agostino and Pearson test (for repeated values)]	Statistical test	95% CI	Statistical statement
a	1*B*; baseline VF	Males: W = 0.8693, *p *=* *0.025; Females: W = 0.7876, *p *=* *0.0019	Mann–Whitney test	[−3.0, −1.0]	*U* = 15, *p *<* *0.0001
b	1*C*; baseline Hargreaves	Males: W = 0.9866, *p *=* *0.9952; Females: W = 0.9324, *p *=* *0.2660	Unpaired Welch’s *t* test	[−1.754, 3.054]	*t*_(23.55)_ = 0.5586, *p *=* *0.5817
c	1*D*; baseline splash latency to groom	Males: W = 0.9147, *p *=* *0.16; Females: W = 0.7800, *p *=* *0.0003	Mann–Whitney test	[−104.6, 204.9]	*U* = 103, *p *=* *0.5140
d	1*E*; baseline splash time spent grooming	Males: W = 0.6525, *p *<* *0.0001; Females: W = 0.8400, *p *=* *0.0097	Mann–Whitney test	[−13.39, 21.96]	*U* = 116, *p *=* *0.8901
e	1*F*; baseline NSF latency to eat	Males: W = 0.8124, *p *=* *0.0040; Females: W = 0.8299, *p *=* *0.0070	Mann–Whitney test	[−90.00, 107.0]	*U* = 119, *p *=* *0.7520
f	1*G*; baseline NSF post-test consumption	Males: W = 0.9618, *p *=* *0.6937; Females: W = 0.9651, *p *=* *0.7552	Student’s *t* test	[−0.7823, 0.8027]	*t*_(30)_ = 0.02634
g	2*A*; EtOH intake	Males: W = 0.9716, *p *=* *0.8644; Females: W = 0.9364, *p *=* *0.3074	Two-way ANOVA	[−2.447, 0.7983]	Day × Time: *F*_(15,207)_ = 1.352, *p *=* *0.1740; Sex: *F*_(1,14)_ = 1.187, *p *=* *0.2943; Time: *F*_(15,207)_ = 1.612, *p *=* *0.0726
h	2*B*; EtOH intake 1 week	Males: W = 0.9635, *p *=* *0.8424; Females: W = 0.9210, *p *=* *0.4830	Student’s *t* test	[2.653, 11.42]	*t*_(14)_ = 3.442, *p *=* *0.0040
i	2*C*; H_2_O intake	Males: W = 0.8956, *p *=* *0.0684; Females: W = 0.9630, *p *=* *0.7156	Two-way ANOVA	[−37.59, 8.638]	Sex × Time: *F*_(15,205)_ = 1.026; *p *=* *0.4302; Sex: *F*_(1,14)_ = 1.804, *p *=* *0.2006; Time: *F*_(15,205)_ = 2.882, *p *=* *0.0004
j	2*D*; H_2_O intake 1 week	Males: W = 0.9289, *p *=* *0.5085; Females: W = 0.8810, *p *=* *0.1924	Student’s *t* test	[−22.85, 218.9]	*t*_(14)_ = 1.739, *p *=* *0.1039
k	2*E*; EtOH preference	Males: W = 0.9617, *p *=* *0.06923; Females: W = 0.9002, *p *=* *0.0811	Two-way ANOVA	[−11.23, 10.85]	Sex × Time: *F*_(15,204)_ = 1.344, *p *=* *0.1784; Sex: *F*_(1,14)_ = 0.001386, *p *=* *0.9708; Time: *F*_(15,204)_ = 3.382, *p *<* *0.0001
l	2*F*; EtOH preference 1 week	Males: W = 0.9040, *p *=* *0.3138; Females: W = 0.8657; *p *=* *0.1368	Student’s *t* test	[0.1420, 12.98]	*t*_(14)_ = 2.192, *p *=* *0.0458
m	2*G*; total fluid intake	Males: W = 0.9242 *p *=* *0.1970; Females: W = 0.9192 *p *=* *0.1638	Two-way ANOVA	[−42.90, −1.558]	Sex × Time: *F*_(15,206)_ = 0.7308, *p *=* *0.7516; Sex: *F*_(1,14)_ = 5.320, *p *=* *0.0369; Time: *F*_(15,206)_ = 1.269, *p *=* *0.2240
n	2*H*; total fluid intake 1 week	Males: W = 0.9372, *p *=* *0.5836; Females: W = 0.9236, *p *=* *0.4599	Welch’s *t* test	[3.221, 282.2]	*t*_(9.424)_ = 2.299, *p *=* *0.0459
o	3*A*; Von Frey	H_2_O Males pre-test: K^2^ = 0.6402, *p *=* *0.7261; H_2_O Females pre-test: K^2^ = 0.6402, *p *=* *0.7261; EtOH Males pre-test: K^2^ = 0.2973, *p *=* *0.8619; EtOH Females pre-test: K^2^ = 1.227, *p *=* *0.5414; H_2_O Males post-test: K2 = 9967, *p *=* *0.6075; H_2_O Females post-test: K^2^ = 2.182, *p *=* *0.3358; EtOH Males post-test: K^2^ = 0.6506, *p *=* *0.7223; EtOH Females post-test: K^2^ = 2.497, *p *=* *0.2870	Three-way ANOVA	NA	Sex × Time × EtOH: *F*_(1,28)_ = 0.000, *p *>* *0.999; Time × EtOH: *F*_(1,28)_ = 5.628, *p *=* *0.0248; Sex × EtOH: *F*_(1,28)_ = 0.04308, *p *=* *0.8371; Sex × Time: *F*_(1,28)_ = 1.674, *p *=* *0.2062; Time: *F*_(1,28)_ = 39.12, *p *<* *0.0001, Sex: *F*_(1,28)_ = 4.308, *p *=* *0.0472; EtOH: *F*_(1,28)_ = 26.92; *p *<* *0.0001
p	3*B*; drinking before and Von Frey	EtOH intake (Males and Females): K^2^ = 2.8323, *p *=* *0.2438; EtOH intake (Males): K^2^ = 0.3602, *p *=* *0.8352; EtOH intake (Females): K^2^ = 3.753, *p *=* *0.1531; Von Frey (Males and Females): K^2^ = 1.085, *p *=* *0.5814; Von Frey (Males): K^2^ = 2.182, *p *=* *0.3358; Von Frey (Females): K^2^ = 2.497, *p *=* *0.2870	Pearson’s correlation	Males and Females: [−0.2136, 0.701] Males: [−0.6831, 0.7250]; Females: [−0.1405, 0.9234]	Males and Females: *r*_(14)_ = 0.3155, *p *=* *0.2340; Males: *r*_(6)_ = 0.04155, *p *=* *0.9222; Females: *r*_(6)_ = 0.6262, *p *=* *0.0967
q	3*C*; Von Frey and drinking after	Von Frey (Males and Females): K^2^ = 1.085, *p *=* *0.5814; Von Frey (Males): K^2^ = 2.182, *p *=* *0.3358; Von Frey (Females): K^2^ = 2.497, *p *=* *0.2870; EtOH intake (Males and Females): K^2^ = 1.487, *p *=* *0.4756; EtOH intake (Males): K^2^ = 0.5575, *p *=* *0.7567; EtOH intake (Females): K^2^ = 1.038, *p *=* *0.5951	Pearson’s correlation	Males and Females: [−0.2646, 0.6730]; Males: [−0.6869, 0.7216]; Females: −[0.2240, 0.9096]	Males and Females: *r*_(14)_ = 0.2660, *p *=* *0.3193, Males: *r*_(6)_ = 0.03441, *p *=* *0.9355; Females: *r*_(6)_ = 0.5708, *p *=* *0.1395
r	3*D*; Hargreaves	H_2_O Males pre-test: K^2^ = 0.5751, *p *=* *0.7501; H_2_O Females pre-test: K^2^ = 0.7428, *p *=* *0.6898; EtOH Males pre-test: K^2^ = 0.07525, *p *=* *0.9631; EtOH Females pre-test: K^2^ = 1.759, *p *=* *0.4149; H_2_O Males post-test: K^2^ = 1.255, *p *=* *0.0.5339; H_2_O Females post-test: K^2^ = 0.05513, *p *=* *0.09728; EtOH Males post-test: K^2^ = 5.464, *p *=* *0.0651; EtOH Females post-test: K^2^ = 5.464, *p *=* *0.0651	Three-way ANOVA	NA	Sex × Time × EtOH: *F*_(1,28)_ = 0.5697, *p *=* *0.4567; Time × EtOH: *F*_(1,28)_ = 3.394, *p *=* *0.0760; Sex × EtOH: *F*_(1,28)_ = 0.2055, *p *=* *0.6538; Sex × Time: *F*_(1,28)_ = 0.2997, *p *=* *0.5884; EtOH: *F*_(1,28)_ = 1.367; *p *=* *0.2521; Sex: *F*_(1,28)_ = 3.529 *p *=* *0.0707; Time: *F*_(1,28)_ = 1.224, *p *=* *0.2780
s	3*E*; drinking before and Hargreaves	EtOH Intake (Males and Females): K^2^ = 2.074, *p *=* *0.3456; EtOH intake (Males): K^2^ = 0.288, *p *=* *0.5251; EtOH intake (Females): K^2^ = 3.507, *p *=* *0.1732; Hargreaves (Males and Females): K^2^ = 4.744, *p *=* *0.0933; Hargreaves (Males): K^2^ = 5.464, *p *=* *0.0651; Hargreaves (Females): K^2^ = 1.582, *p *=* *0.4534	Pearson’s correlation followed by Fisher’s *r* to *z* transformation	Males and Females: [−0.2458, 0.6838]; Males: [0.5502, 0.9827]; Females: − [0.7679, 0.6278]	Pearson’s: Males and Females: *r*_(14)_ = 0.2846, *p *=* *0.2853; Males: *r*_(6)_ = 0.9043, *p *=* *0.0020; Females: *r*_(6)_ = −0.1378, *p *=* *0.7488 Fishers *r* to *z*: *z*_obs_ = 2.5836, *p *=* *0.0098
t	3*F*; Hargreaves and drinking after	Hargreaves (Males and Females): K^2^ = 4.744, *p *=* *0.0933; Hargreaves (Males): K^2^ = 5.464, *p *=* *0.0651; Hargreaves (Females): K^2^ = 1.582, *p *=* *0.4534; EtOH Intake (Males and Females): K^2^ = 1.758, *p *=* *0.4152; EtOH Intake (Males): K^2^ = 0.5217, *p *=* *0.7704, EtOH intake (Females): K^2^ = 2.740, *p *=* *0.2541	Pearson’s correlation followed by Fisher’s *r* to *z* transformation	Males and Females: [−0.5232, 0.4672]; Males: [0.3605, 0.9722]; Females: −[0.9226, 0.1460]	Pearson’s: Males and Females together: *r*_(14)_ = −0.03709, *p *=* *0.8915; Males: *r*_(6)_ = 0.8494, *p *=* *0.0075; Females: *r*_(6)_ = −0.6227, *p *=* *0.0991 Fisher’s *r* to *z*; *z*_obs_ = 3.1360, *p *=* *0.0017
u	4*A*; splash test: latency to groom	H_2_O Males pre-test: W = 0.8928, *p *=* *0.3121; H_2_O Females pre-test: K^2^ = 2.030, *p *=* *0.3624; EtOH Males pre-test: K^2^ = 1.950, *p *=* *0.3773; EtOH Females pre-test: K2 = 3.028, *p *=* *0.2200; H_2_O Males post-test: W = 0.9594, *p *=* *0.9138; H_2_O Females post-test: K^2^ = 4.792, *p *=* *0.0911; EtOH Males post-test: K^2^ = 2.392, *p *=* *0.3025; EtOH Female post-test: K^2^ = 3.433, *p *=* *01797	Three-way ANOVA	NA	Sex × Time × EtOH: *F*_(1,27)_ = 0.03307, *p *=* *0.8570; Time × EtOH: *F*_(1,27)_ = 0.3165, *p *=* *0.5784; Sex × EtOH: *F*_(1,27)_ = 4.164, *p *=* *0.0512; Sex × Time: *F*_(1,27)_ = 2.776, *p *=* *0.1073; EtOH: *F*_(1,27)_ = 0.6438; *p *=* *0.5923; Sex: *F*_(1,27)_ = 3.782, *p *=* *0.0623; Time: *F*_(1,27)_ = 0.9851, *p *=* *0.3298
v	4*B*: splash test: time spent grooming	H_2_O Males pre-test: W = 0.9455, *p *=* *0.5887; H_2_O Females pre-test: K^2^ = 1.531, *p *=* *0.4652; EtOH Males pre-test: K^2^ = 13.46, *p *=* *0.0012; EtOH Females pre-test: K^2^ = 4.000, *p *=* *0.153; H_2_O Males post-test: W = 0.8747, *p *=* *0.2038; H_2_O females post-test: K^2^ = 3.047, *p *=* *0.2179; EtOH-Males post-test: K^2^ = 4.314, *p *=* *0.1156; EtOH Females post-test: K^2^ = 2.577, *p *=* *0.2756	Three-way ANOVA	NA	Sex × Time × EtOH: *F*_(1,27)_ = 2.150, *p *=* *0.1551; Time × EtOH: *F*_(1,27)_ = 0.1993, *p *=* *0.6589; Sex × EtOH: *F*_(1,27)_ = 1.272, *p *=* *0.2694; Sex × Time: *F*_(1,27)_ = 0.07479, *p *=* *0.7866; EtOH: *F*_(1,27)_ = 0.1483e-007; *p *=* *0.9997; Sex: *F*_(1,27)_ = 0.03729, *p *=* *0.8483; Time: *F*_(1,27)_ = 4.743, *p *=* *0.0383
w	4*C*; cumulative incidence (pre-test)	NA	Mantel–Cox log-rank	NA	χ² = 4.469, df = 3, *p *=* *0.2151
x	4*D*; cumulative incidence (post-test)	NA	Mantel–Cox log-rank test followed by Bonferroni correction	NA	χ² = 6.968, df = 3, *p *=* *0.0467
y	4*E*; drinking before and latency to groom	EtOH intake (Males and Females): K^2^ = 1.986, *p *=* *0.3704; EtOH intake (Males): K^2^ = 0.7800, *p *=* *0.6671; EtOH intake (Females): K^2^ = 0.7666, *p *=* *0.6816; Splash Latency (Males and Females): K^2^ = 2.543, *p *=* *0.2804; splash latency (Males): K^2^ = 2.392, *p *=* *0.3025; splash latency (Females): K^2^ = 3.433, *p *=* *0.1797	Pearson’s correlation	Males and Females: [0.1649, 0.8493]; Males: [−0.4124, 0.8654]; Females: [−0.1547, 0.9212]	Males and Females together: *r*_(14)_ = 0.6108, *p *=* *0.0120; Males: *r*_(6)_ = 0.4120, *p *=* *0.3105; Females: *r*_(6)_ = 0.6172, *p *=* *0.1030
z	4*F*; latency to groom and drinking after	splash latency (Males and Females): K^2^ = 3.211, *p *=* *0.2008; splash latency (Males): K^2^ = 2.392, *p *=* *0.3205; splash latency (Females): *n* too small; EtOH intake (Males and Females): K^2^ = 2.408, *p *=* *0.3000; EtOH intake (Males): K^2^ = 0.5217, *p *=* *0.7704; EtOH intake (Females): *n* too small	Pearson’s correlation	Males and Females: [−0.2918, 0.6811]; Males: [−0.3425, 0.8845]; Females: [−0.7008, 0.7973]	Males and Females together: *r*_(13)_ = 0.2592, *p *=* *0.3508; Males: *r*_(6)_ = 0.4774, *p *=* *0.2269; Females: *r*_(5)_ = 0.1107, *p *=* *0.3132
aa	4*G*; drinking before and time spent grooming	EtOH intake (Males and Females): K^2^ = 1.986, *p *=* *0.3704; EtOH intake (Males): K^2^ = 0.7800, *p *=* *0.6671; EtOH intake (Females): K^2^ = 0.7666, *p *=* *0.6816; splash grooming (Males and Females): K^2^ = 8.434, *p *=* *0.0147; splash grooming (Males): K^2^ = 4.314, *p *=* *0.1156; splash grooming (Females): K^2^ = 2.577, *p *=* *0.2756	Spearman’s correlation	Males and Females: [−0.8429, −0.1112]; Males: *n* too small; Females: *n* too small	Males and Females: *r*_s(14)_ = −0.5859, *p *=* *0.0193; Males: *r*_s(6)_ = −0.8456, *p *=* *0.0179; Females: *r*_s(6)_ = 0.07143, *p *=* *0.0179
ab	4*H*; time spent grooming and drinking after	splash grooming (Males and Females): K^2^ = 15.70, *p *=* *0.0004, splash grooming (Males): K^2^ = 4.314, *p *=* *0.1156; splash grooming (Females): *n* too small; EtOH intake (Males and Females): K^2^ = 2.408, *p *=* *0.3000; EtOH intake (Males): K^2^ = 0.5217, *p *=* *0.7704; EtOH intake (Females): *n* too small	Spearman’s correlation	Males and Females: [−0.5456, 0.5028]; Males: *n* too small; Females: *n* too small	Males and Females: *r*_s(13)_ = −0.02951, *p *=* *0.9180; Males: *r*_s(6)_ = 0.09524, *p *=* *0.8401; Females: *r*_s(5)_ = −0.1782, *p *=* *0.7182
ac	5*A*; NSF latency	On log-transformed values: H_2_O Males pre-test: K^2^ = 1.272, *p *=* *0.5293; H_2_O Females pre-test: K^2^ = 0.8888, *p *=* *0.6412; EtOH Males pre-test: K^2^ = 1.481, *p *=* *0.4769; EtOH Females pre-test: K^2^ = 4.336, *p *=* *0.1144; H_2_O Males post-test: K^2^ = 1.460, *p *=* *0.4819; H_2_O Females post-test: K^2^ = 1.083, *p *=* *0.5819; EtOH Males post-test: K^2^ = 1.342, *p *=* *0.5112; EtOH Females post-test: K^2^ = 2.836, *p *=* *0.2421	Three-way ANOVA	NA	Sex × Time × EtOH: *F*_(1,28)_ = 0.09325, *p* = 0.7623; Time × EtOH: *F*_(1,28)_ = 0.01534, *p *=* *0.9023; Sex × EtOH: *F*_(1,28)_ = 0.9187, *p *=* *0.3460; Sex × Time: *F*_(1,28)_ = 1.512, *p *=* *0.2291; EtOH: *F*_(1,28)_ = 0.3362; *p *=* *0.5667; Sex: *F*_(1,28)_ = 0.4367, *p *=* *0.5141; Time: *F*_(1,28)_ = 7.069, *p *=* *0.0128
ad	5*B*; NSF post-test consumption	H_2_O Males pre-test: K^2^ = 0.9742, *p *=* *0.6144; H_2_O Females pre-test: K^2^ = 0.7841, *p *=* *0.6757; EtOH Males pre-test: K^2^ = 0.2186, *p *=* *0.989; EtOH Females pre-test: K^2^ = 0.09536, *p *=* *0.9534; H_2_O Males post-test: K^2^ = 2.543, *p *=* *0.2805; H_2_O Females post-test: K^2^ = 1.92, *p *=* *0.3786; EtOH Males post-test: K^2^ = 0.175, *p *=* *0.9289; EtOH Females post-test: K^2^ = 0.1529, *p *=* *0.9264	Three-way ANOVA	NA	Sex × Time × EtOH: *F*_(1,28)_ = 0.2294, *p *=* *0.6357; Time × EtOH: *F*_(1,28)_ = 0.1.494, *p *=* *0.2317; Sex × EtOH: *F*_(1,28)_ = 0.8227, *p *=* *0.3727; Sex × Time: *F*_(1,28)_ = 0.001506, *p *=* *0.9693; EtOH: *F*_(1,28)_ = 1.248; *p *=* *0.2734; Sex: *F*_(1,28)_ = 0.006310, *p *=* *0.9372; Time: *F*_(1,28)_ = 29.96 *p *<* *0.0001
ae	5*C*; drinking before and NSF latency	EtOH intake (Males and Females): K^2^ = 2.823, *p *=* *0.2438; EtOH intake (Males) = 0.3602, *p *=* *0.8352; EtOH intake (Females): K^2^ = 3.751, *p *=* *0.1531; NSF Latency (Males and Females): K^2^ = 18.87, *p *<* *0.0001; NSF latency (Males): K^2^ = 5.519, *p *=* *0.0633; NSF Latency (Females): K^2^ = 21.53; *p *<* *0.0001	Spearman’s correlation	Males and Females: [0.07988, 0.8335]; Males: *n* too small; Females: *n* too small	Males and Females: *r*_s(14)_ = 0.5647, *p *=* *0.0248; Males: *r*_s(6)_ = 0.7381, *p *=* *0.0458; Females: *r*_s(6)_ = 0.4286, *p *=* *0.2992
af	5*D*; NSF latency and drinking after	NSF Latency (Males and Females): K^2^ = 18.87, *p *<* *0.0001; NSF latency (Males): K^2^ = 5.519, *p *=* *0.0633; NSF Latency (Females): K^2^ = 21.53; *p *<* *0.0001; EtOH intake (Males and Females): K^2^ = 1.068, *p *=* *0.5862; EtOH intake (Males): K^2^ = 1.074, *p *=* *0.5844; EtOH intake (Females): K^2^ = 0.3717, *p *=* *0.8304	Spearman’s correlation	Males and Females: [−0.3506, 0.6371]; Males: *n* too small; Females: *n* too small	Males and Females: *r*_s(14)_ = 0.1912, *p *=* *0.4769; Males: *r*_s(6)_ = 0.6667, *p *=* *0.0831; Females: *r*_s(6)_ = −0.1905 *p *=* *0.6646
ag	5*E*; drinking before and NSF post-test consumption	EtOH intake (Males and Females): K^2^ = 2.823, *p *=* *0.2438; EtOH intake (Males) = 0.3602, *p *=* *0.8352; EtOH intake (Females): K^2^ = 3.751, *p *=* *0.1531; NSF post-test (Males and Females): K^2^ = 0.4805, *p *=* *0.7864; NSF post-test (Males): K^2^ = 0.1475, *p *=* *0.9289; NSF post-test (Females): K^2^ = 14.75, *p *=* *0.2438	Pearson’s correlation	Males and Females: [−0.09538, 0.7580]; Males: [−0.6119, 0.7783]; Females: [−0.04114, 0.9369]	Males and Females: *r*_(14)_ = 0.4202, *p *=* *0.1051; Males: *r*_(6)_ = 0.1630, *p *=* *0.6997; Females: *r*_(6)_ = −0.6833 *p *=* *0.0617
ah	5*F*; NSF post-test consumption and drinking after	NSF post-test (Males and Females: K^2^ = 0.4805, *p *=* *0.7864; NSF post-test (Males): K^2^ = 0.1475, *p *=* *0.9289; NSF post-test (Females): K^2^ = 14.75, *p *=* *0.2438; EtOH intake (Males and Females): K^2^ = 1.068, *p *=* *0.5862; EtOH intake (Males): K^2^ = 1.074, *p *=* *0.5844; EtOH intake (Females): K^2^ = 0.3717, *p *=* *0.8304	Pearson’s correlation	Males and Females: [−0.3398, 0.6251]; Males: [−0.7631, 0.6349]; Females: [−0.3945, 0.8707]	Males and Females: *r*_(14)_ = 0.1875, *p *=* *0.4867; Males: *r*_(6)_ = −0.1263, *p *=* *0.7657; Females: *r*_(6)_ = 0.4296 *p *=* *0.2881
ai	6*B*; prelimbic CRF1+ cells	H_2_O Males: W = 0.9387, *p *=* *0.5982; EtOH Males: W = 0.8026, *p *=* *0.0305; H_2_O Females: W = 0.9307, *p *=* *0.5227; EtOH Females: W = 0.9863; *p *=* *0.9871	Two-way ANOVA	[−98.40, 66.86]	Sex × EtOH: *F*_(1,28)_ = 0.0005307, *p *=* *0.9818; EtOH: *F*_(1,28)_ = 0.002376, *p *=* *0.9615; Sex: *F*_(1,28)_ = 0.1528, *p *=* *0.6988
aj	6*C*; prelimbic cFos+ cells	H_2_O Males: W = 0.9863, *p *=* *0.9871; EtOH Males: W = 0.9250, *p *=* *0.4715; H_2_O Females: W = 0.8285, *p *=* *0.0573; EtOH Females: W = 0.2578; *p *=* *0.1403	Two-way ANOVA	[−413.1, 70.23]	Sex × EtOH: *F*_(1,28)_ = 0.0003888, *p *=* *0.9844; EtOH: *F*_(1,28)_ = 3.972, *p *=* *0.0561; Sex: *F*_(1,28)_ = 2.112 *p *=* *0.1573
ak	6*D*; prelimbic total double labeled cells	H_2_O Males: W = 0.8668, *p *=* *0.1403; EtOH Males: W = 0.9094, *p *=* *0.3501; H_2_O Females: W = 0.8506, *p *=* *0.0966; EtOH Females: W = 0.9591; *p *=* *0.8018	Two-way ANOVA	[−46.90, 5.043]	Sex × EtOH: *F*_(1,28)_ = 0.1194, *p *=* *0.7323; EtOH: *F*_(1,28)_ = 1.108, *p *=* *0.3015; Sex: *F*_(1,28)_ = 2.725 *p *=* *0.1100
al	6*E*; prelimbic double labeled (%CRF1 cells)	H_2_O Males: W = 0.9421, *p *=* *0.6316; EtOH Males: W = 0.9044, *p *=* *0.3165; H_2_O Females: W = 0.9501, *p *=* *0.7127; EtOH Females: W = 0.9399; *p *=* *0.61	Two-way ANOVA	[−11.99, 0.1321]	Sex × EtOH: *F*_(1,28)_ = 0.1477, *p *=* *0.7037; EtOH: *F*_(1,28)_ = 2.757, *p *=* *0.1080; Sex: *F*_(1,28)_ = 4.015 *p *=* *0.0549),
am	6*G*; infralimbic CRF1+ cells	H_2_O Males: W = 0.9538, *p *=* *0.7494; EtOH Males: W = 0.9358, *p *=* *0.57; H_2_O Females: W = 0.9501, *p *=* *0.7125; EtOH Females: W = 0.9718, *p *=* *0.9121	Two-way ANOVA	[−7.164, 19.40]	Sex × EtOH: *F*_(1,28)_ = 0.7886, *p *=* *0.3821; EtOH: *F*_(1,28)_ = 0.01444, *p *=* *0.9052; Sex: *F*_(1,28)_ = 0.8904, *p *=* *0.3534
an	6*H*; infralimbic cFos+ cells	H_2_O Males: W = 0.9011, *p *=* *0.2954; EtOH Males: W = 0.9654, *p *=* *0.8593; H_2_O Females: W = 0.9354, *p *=* *0.5661; EtOH Females: W = 0.8903, *p *=* *0.2355	Two-way ANOVA	[−104.3, −14.12]	Sex × EtOH: *F*_(1,28)_ = 0.000825, *p *=* *0.9773; EtOH: *F*_(1,28)_ = 1.639, *p *=* *0.2110; Sex: *F*_(1,28)_ = 7.233, *p *=* *0.0119
ao	6*I*; infralimbic double labeled cells (total)	H_2_O Males: W = 0.9146, *p *=* *0.3874; EtOH Males: W = 0.9709, *p *=* *0.9052; H_2_O Females: W = 0.9709, *p *=* *0.9052; EtOH Females: W = 0.8742, *p *=* *0.1657	Two-way ANOVA	[−3.729, 1.057]	Sex × EtOH: *F*_(1,28)_ = 2.961, *p *=* *0.0963; EtOH: *F*_(1,28)_ = 2.026, *p *=* *0.1657; Sex: *F*_(1,28)_ = 1.307, *p *=* *0.2626
ap	6*J*; infralimbic double labeled (% CRF1+)	H_2_O Males: W = 0.9810, *p *=* *0.9679; EtOH Males: W = 0.9193, *p *=* *0.4240; H_2_O Females: W = 0.9799, *p *=* *0.9625; EtOH Females: W = 0.9043, *p *=* *0.3159	Two-way ANOVA	[−2.276, 7.709]	Sex × EtOH: *F*_(1,28)_ = 0.7972, *p *=* *0.3795; EtOH: *F*_(1,28)_ = 1.242, *p *=* *0.2746; Sex: *F*_(1,28)_ = 7.356, *p *=* *0.0113
aq	6*K*; infralimbic CRF1+ correlations with EtOH intake	EtOH intake (Males and Females): W = 0.9551, *p *=* *0.5745; EtOH Intake (Males): W = 0.9177, *p *=* *0.4116; EtOH Intake (Females): W = 0.9610, *p *=* *8197; IHC (Males and Females): W = 0.9490, *p *=* *0.4744; IHC (Males): W = 0.9358, *p *=* *0.57; IHC (Females): W = 0.9718, *p *=* *0.9121	Pearson’s correlation followed by Fisher’s *r* to *z* transformation	Males and Females: [−0.5537, 0.4329]; Males: [−0.9468, −0.04716]; Females: [0.3041, 0.9685]	Pearson’s: Males and Females: *r*_(14)_ = −0.07998, *p *=* *0.7684; Males: *r*_(6)_ = −0.7277, *p *=* *0.0407; Females: *r*_(6)_ = 0.8307, *p *=* *0.0106; Fisher’s *r* to *z*: *z*_obs_ = −3.3429, *p *=* *0.0008
ar	6*L*; infralimbic cFos+ correlations with EtOH intake	EtOH intake (Males and Females): W = 0.9551, *p *=* *0.5745; EtOH Intake (Males): W = 0.9177, *p *=* *0.4116; EtOH Intake (Females): W = 0.9610, *p *=* *8197; IHC (Males and Females): W = 0.9130, *p *=* *0.1310, IHC (Males): W = 0.9654, *p *=* *0.8593; IHC (Females): W = 0.9654, *p *=* *0.8593	Pearson’s correlation followed by Fisher’s *r* to *z* transformation	Males and Females: [−0.6137, 0.3561]; Males: [−0.9576, −0.1612]; Females: [−0.5743, 0.8002]	Pearson’s: Males and Females: *r*_(14)_ = −0.1695, *p *=* *0.5403; Males: *r*_(6)_ = −0.7775, *p *=* *0.0231; Females: *r*_(6)_ = 0.2192, *p *=* *0.6022 Fisher’s *r* to *z*: *z*_obs_ = −1.19950, *p *=* *0.0460
as	6*M*; infralimbic total double labeled correlations with EtOH intake	EtOH intake (Males and Females): W = 0.9551, *p *=* *0.5745; EtOH Intake (Males): W = 0.9177, *p *=* *0.4116; EtOH Intake (Females): W = 0.9610, *p *=* *8197; IHC (Males and Females): W = 0.9678, *p *=* *0.8015; IHC (Males): W = 0.9311, *p *=* *0.5265; IHC (Females): W = 0.8742, *p *=* *0.1657	Pearson’s correlation followed by Fisher’s *r* to *z* transformation	Males and Females: [−0.6273, 0.3366]; Males: [−0.9571, −0.1554]; Females: [−0.2515, 0.9044]	Pearson’s: Males and Females: *r*_(14)_ = −0.3485, *p *=* *0.1858; Males: *r*_(6)_ = −0.5444, *p *=* *0.1630; Females: *r*_(6)_ = −0.4090, *p *=* *0.3144 Fisher’s *r* to *z*: *z*_obs_ = −2.6132, *p *=* *0.0090
at	6*N*; infralimbic double labeled (%CRF1+) correlations with EtOH intake	EtOH intake (Males and Females): W = 0.9551, *p *=* *0.5745; EtOH Intake (Males): W = 0.9177, *p *=* *0.4116; EtOH Intake (Females): W = 0.9610, *p *=* *8197; IHC (Males and Females): W = 0.8705, *p *=* *0.0277; IHC (Males): W = 0.9193, *p *=* *0.4240; IHC (Females): W = 0.9043, *p *=* *0.3159	Pearson’s correlation	Males and Females: [−0.7199, 0.1779]; Males: [−0.9027, 0.2601]; Females: [−0.8645, 0.4154]	Males and Females: *r*_(14)_ = −0.3485, *p *=* *0.1858; Males: *r*_(6)_ = −0.5444, *p *=* *0.1630; Females: *r*_(6)_ = −0.4090, *p *=* *0.3144

Baseline behavioral tests were analyzed using an unpaired Student’s *t* test (parametric data with equal sample variances), Welch’s *t* test (parametric data with unequal sample variances), or a Mann–Whitney *U* test (nonparametric data). For cumulative incidence graphs, Kaplan–Meier survival curves were generated and were followed by Mantel–Cox log-rank test (followed by Bonferroni when applicable). Fluid intake and preference were analyzed by a mixed model two-way ANOVA, using session as a within-subjects factor and sex as a between-subjects factor. Cumulative intake data were analyzed using an unpaired Student’s *t* test (equal sample variances) or an unpaired Welch’s *t* test (unequal sample variances). When behavioral tests were repeated (following drinking), data were analyzed using a three-way mixed ANOVA (using time as a within-subjects factor while EtOH and sex were analyzed as between-subjects factors). When applicable, Bonferroni’s correction was used for multiple comparisons. Correlational analyses were performed using Pearson’s correlation (parametric data) or Spearman’s correlation (nonparametric data). To compare correlations between males and females, Fisher’s *r* to *z* transformation was used:

$$\text{Fisher}’\text{s Z}:{\text{ z}}_{\text{r}}=0.5l\text{n}\left(\left(\text{1}+\text{r}\right)/\left(\text{1}-\text{r}\right)\right)\text{ where r }=\text{ Pearson}’\text{s r }\left(\text{or Spearman}’{\text{s r}}_{\text{s}}\right).$$

Z_r_ from males and females (denoted z_m_ and z_f_, respectively) was used to compute an observed z value, z_obs_ with the following calculation:

$${\text{z}}_{\text{obs}}=\frac{{\text{z}}_{\text{m}}-{\text{z}}_{\text{f}}}{\text{sqrt}\left(\text{1}/\left({\text{N}}_{\text{m}}-\text{3}\right)+\text{1}/\left({\text{N}}_{\text{f}}-\text{3}\right)\right)},$$

where N = number of males (N_m_) or females (N_f_).

In each set of correlations, the same analysis (Pearson’s or Spearman’s) was used for both sexes to calculate a z_obs_.

Immunofluorescence data were analyzed by a two-way ANOVA using sex and EtOH as between-subjects factors.

## Results

### Experimental timeline

**Figure 1.**
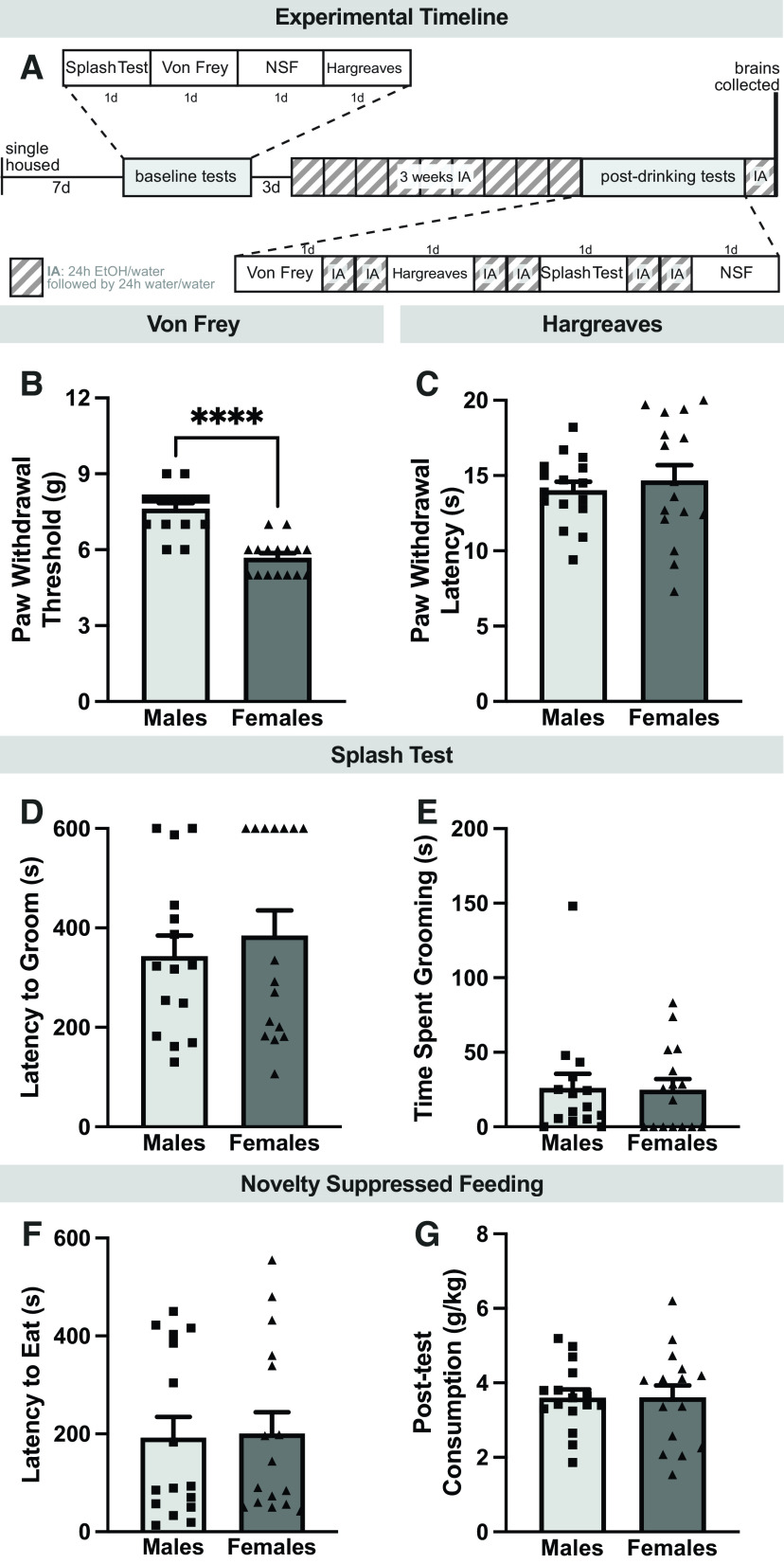
Experimental timeline and baseline behavior. ***A***, Timeline of entire experiment. ***B***, Mechanical sensitivity in Von Frey test, as measured by paw withdrawal threshold. ***C***, Thermal sensitivity in the Hargreaves test, as measured by latency to withdraw. Affective behavior as measured by latency to groom (***D***) and time spent grooming (***E***). Cumulative incidence of grooming initiation is shown in Extended Data Figure 1-1. ***F***, Affective behavior in the novelty suppressed feeding test, as measured by latency to eat. ***G***, Appetitive behavior in the novelty suppressed feeding test, as measured by post-test consumption. Cumulative incidence of feeding initiation is shown in Extended Data Figure 1-2. ****p <* *0.0001 (Mann–Whitney test). Data are expressed as individual points with bars representing mean + SEM.

As shown in Figure 1*A*, rats were single-housed for one week before the onset of behavioral tests, which were conducted on separate but consecutive days. After baseline testing was complete, rats were left undisturbed for 3 d before being split into two groups, one allowed access to a bottle containing water and a bottle containing 20% EtOH under an intermittent access paradigm, and the other allowed access to only water bottles. After three weeks of drinking, postdrinking behavioral testing began. These tests were separated by at least two drinking sessions to ensure testing did not disrupt EtOH consumption. During each test, rats were run in a pseudo-randomized subject order to minimize effects of circadian cycle and withdrawal time. After the last behavioral test, rats underwent a final EtOH drinking session. Twenty-four hours after the final drinking session, rats were perfused and tissue was collected as described above.

10.1523/ENEURO.0055-23.2023.f1-1 Extended Data Figure 1-1 Cumulative incidence of grooming initiation in the splash test. Even when accounting for the rats that never groomed (rather than artificially assigning them a maximum value or excluding them from studies), there was no sex difference in grooming initiation (Mantel–Cox log-rank test: χ^2^ = 1.430, df = 1, *p *=* *0.2318). There was also no sex difference in total time spent grooming (***C***; Mann–Whitney test, *p *=* *0.8901). Download Figure 1-1, EPS file.

**Figure 2.**
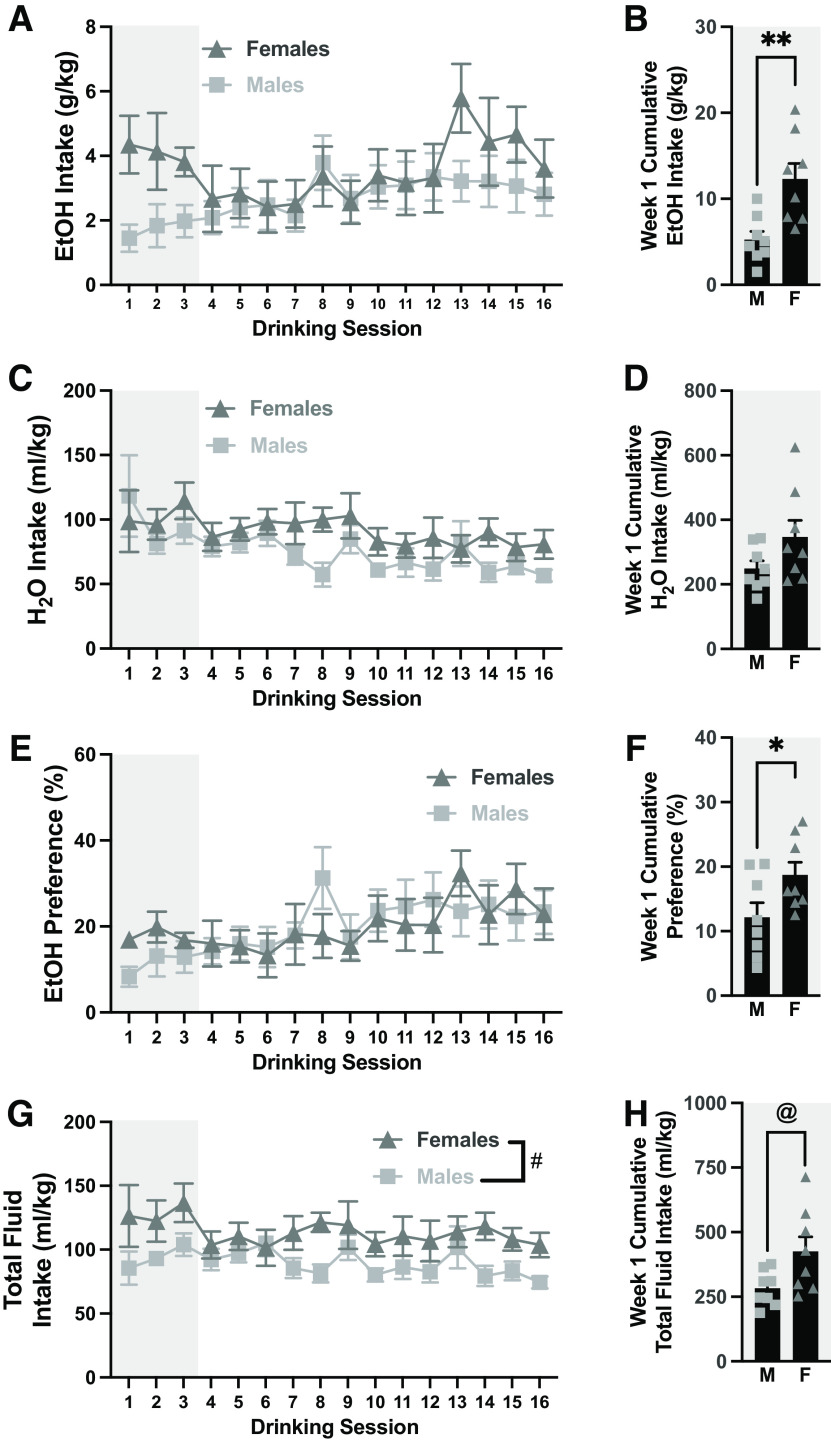
Voluntary EtOH drinking under an intermittent access paradigm. ***A***, EtOH intake over time. ***B***, Cumulative EtOH intake during the first week of EtOH access. ***C***, Water intake over time. ***D***, Cumulative water intake during the first week of EtOH access. ***E***, EtOH preference over time. ***F***, Cumulative EtOH preference during the first week of EtOH access. ***G***, Total fluid intake over time. ***H***, Cumulative total fluid intake during the first week of EtOH access. Relationship between baseline nociceptive and affective behavior is shown in Extended Data Figure 2-1. **p <* *0.01, *p <* *0.01 (unpaired Student’s *t* test); &*p *<* *0.05 (two-way ANOVA, main effect of sex); @*p *<* *0.05 (unpaired Welch’s *t* test). Data are expressed as individual points (when feasible) with bars representing mean + SEM.

10.1523/ENEURO.0055-23.2023.f1-2 Extended Data Figure 1-2 Cumulative incidence of feeding initiation in the novelty suppressed feeding test. There was no effect of sex on cumulative occurrence of feeding initiation (***B***; Mantel–Cox log-rank test, χ^2^ = 0.3366, df = 1, *p *=* *0.5618). Download Figure 1-2, EPS file.

### Baseline affective and nociceptive behavior

Rats underwent a series of baseline behavioral tests to probe potential sex differences in behavior before EtOH drinking. In particular, we found sex differences in the Von Frey test of mechanosensitivity where female rats exhibited decreased withdrawal thresholds^a^, suggesting increased mechanical sensitivity (Fig. 1*B*). However, these differences were unique to mechanosensation, as there were no sex differences in the Hargreaves test of thermosensitivity^b^ (Fig. 1C). Additionally, there were no sex differences in the splash test parameters including latency to groom^c^ (Fig. 1*D*) or time spent grooming^d^ (Fig. 1*E*). Some animals never initiated grooming and were thus assigned artificial latency values of 600 s; however, cumulative incidence of grooming initiation curves (Extended Data Fig. 1-1) revealed no differences between sexes. There were also no sex differences in latency to eat^e^ (Fig. 1*F*; log-transformed in Extended Data Fig. 1-2*A*) or post-test home-cage chow consumption^f^ (Fig. 1*G*) in the NSF test. Similarly, there were no differences in cumulative initiation of feeding (Extended Data Fig. 1-2*B*). Together, these results indicate that while female CRF1-cre rats display increased basal mechanosensitvity, there are no sex differences in the splash test or NSF.

### Voluntary EtOH drinking

After assessing baseline affective behavior, rats were divided into two groups. The rats in the EtOH group were presented with 20% EtOH and water under an intermittent access two-bottle-choice procedure, whereas the rats in the water group received two water bottles. While there were no significant sex differences in overall EtOH intake^g^ (Fig. 2*A*); females consumed significantly more EtOH in the first week of drinking^h^ (Fig. 2*B*). Interestingly, there were no sex differences in water intake overall^i^ (Fig. 2*C*) or during the first week^j^ (Fig. 2*D*), but water intake decreased over time in both sexes. We also looked at EtOH preference, calculated as volume of 20% EtOH consumed divided by volume of total fluid consumed during the session × 100%. While EtOH preference increased over time in a nonsex-dependent manner^k^ (Fig. 2*E*), females exhibited a stronger EtOH preference during the first week^l^ (Fig. 2*F*). Additionally, females consumed significantly more total fluid compared with males overall^m^ (Fig. 2*G*) and during the first week^n^ (Fig. 2*H*). There were no significant correlations between any of the nociceptive or affective behaviors tested and initial EtOH intake (Extended Data Fig. 2-1).

10.1523/ENEURO.0055-23.2023.f2-1 Extended Data Figure 2-1 Behavioral measures did not correlate with first day EtOH intake in both males (M) and females (F). There was a trend where the rats with lower withdrawal thresholds drank more EtOH (***A***). Interestingly, there was no association between initial EtOH intake and basal thermal withdrawal (***B***), or basal latency to groom (***C***) and time spent grooming (***D***) in the splash test. There was also no significant relationship between initial EtOH intake and latency to eat (***E***) or post-test consumption (***F***) in the NSF test. Pearson’s correlations: ***A***, M&F: *r*_(14)_ = −0.4742, *p *=* *0.0635; Males: *r*_(6)_ = 0.5673, *p *=* *0.1425; Females: *r*_(6)_ = −0.2699, *p *=* *0.5179; ***B***, M&F *r*_(14)_ = 0.1836, *p *=* *0.4962; Males: *r*_(6)_ = 0.3344, *p *=* *0.4162; Females: *r*_(6)_ = 0.1388 *p *=* *0.7431; ***F***, M&F: *r*_(14)_ = 0.1751, *p *=* *0.5167; Males: *r*_(6)_ = 0.4654, *p *=* *0.2451; Females: *r*_(6)_ = −0.01082, *p *=* *0.9797. Spearman’s correlations: ***C***, M&F: *r*_s(14)_ = 0.3991, *p *=* *0.1266; Males: *r*_s(6)_ = 0.03593, *p *=* *0.9434; Females: *r*_s(6)_ = 0.4637, *p *=* *0.2619; ***D***, M&F together: *r*_s(14)_ = −0.2088, *p *=* *0.4355, Males: *r*_s(6)_ = 0.3234, *p *=* *0.4317; Females: *r*_s(6)_ = −0.4364, *p *=* *0.3036; ***E***, M&F: *r*_s(14)_ = 0.1177, *p *=* *0.6631; Males: *r*_s(6)_ = 0.3571; *p *=* *0.3894; Females: *r*_s(6)_ = −0.2857, *p *=* *0.5008 Download Figure 2-1, EPS file.

### EtOH drinking and associated pain sensitivity

**Figure 3.**
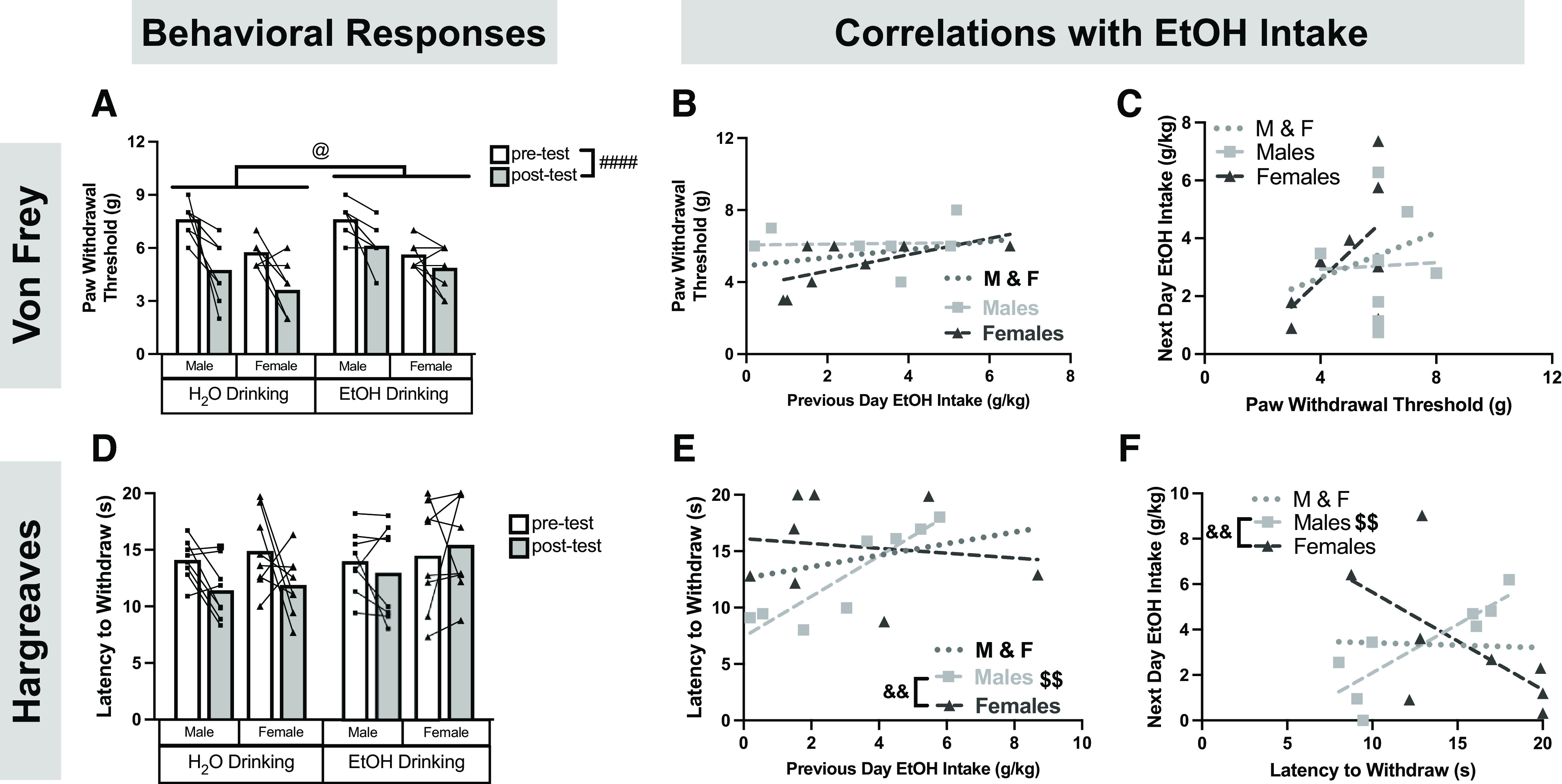
Mechanical and thermal sensitivity following chronic EtOH drinking in males and females. The Von Frey test was repeated and mechanical sensitivity (***A***) was correlated with previous day EtOH intake (***B***) and subsequent EtOH intake (***C***). The Hargreaves test was also repeated and thermal sensitivity (***D***) was correlated with previous day EtOH intake (***E***) and subsequent EtOH intake (***F***). Nociceptive testing did not affect EtOH intake, as demonstrated in Extended Data Figure 3-1. ####*p *<* *0.0001 (three-way ANOVA, main effect of time); @*p *<* *0.05 (three-way ANOVA, main effect of EtOH); $$*p *<* *0.01 (Pearson’s correlation); &&*p *<* *0.01 (Fisher’s *r* to *z* transformation). Data are presented as individual points. When applicable, bars represent mean. Pearson’s correlations: ***B***, M&F: *r*_(14)_ = 0.3155, *p *=* *0.2340; Males: *r*_(6)_ = 0.04,155, *p *=* *0.9222; Females: *r*_(6)_ = 0.6262, *p *=* *0.0967; ***C***, M&F: *r*_(14)_ = 0.2660, *p *=* *0.3193; Males: *r*_(6)_ = 0.03,441, *p *=* *0.9355; Females: *r*_(6)_ = 0.5708, *p *=* *0.1395; ***E***, M&F: *r*_(14)_ = 0.2846, *p *=* *0.2853; Males: *r*_(6)_ = 0.9043, *p *=* *0.0020; Females: *r*_(6)_ = −0.1378, *p *=* *0.7448; ***F***, M&F: *r*_(14)_ = −0.03,709, *p *=* *0.8915; Males: *r*_(6)_ = 0.8494, *p *=* *0.0075; Females: *r*_(6)_ = −0.6227, *p *=* *0.0991.

After three weeks of EtOH access, the rats began postdrinking behavioral testing beginning with the Von Frey test of mechanosensitivity. There was a main effect of time, where paw withdrawal threshold decreased in all four groups^o^ (Fig. 3*A*), suggesting that all rats became more sensitive to mechanical stimuli over time. A main effect of EtOH^o^ also emerged, where EtOH-drinking rats had increased paw withdrawal thresholds, indicative of decreased mechanical sensitivity. Furthermore, there was an EtOH by time interaction, where Bonferroni *post hoc* analysis revealed that the decrease in paw withdrawal threshold over time was significantly stronger in water drinking rats compared with EtOH-drinking rats. Bonferroni *post hoc* analysis also indicated that the main effect of EtOH was driven predominantly by the post-test rather than pre-test, as there was no significant difference between water and EtOH-drinking rats at baseline. Finally, there was a main effect of sex^o^, where females exhibited decreased paw withdrawal thresholds suggesting increased mechanical sensitivity (consistent with Fig. 1*B*). Interestingly, paw withdrawal threshold did not correlate with previous day^p^ (Fig. 3*B*) or subsequent day EtOH intake^q^ (Fig. 3*C*).

10.1523/ENEURO.0055-23.2023.f3-1 Extended Data Figure 3-1 Pain testing does not affect EtOH intake. There was no effect of Von Frey testing (***A***) or Hargreaves testing (***B***) on EtOH consumption. Two-way ANOVAs: ***A***, Sex × Day: *F*_(1,14)_ = 0.1799, *p *=* *0.6779; Sex: *F*_(1,14)_ = 0.01875, *p *=* *0.8930; Day: *F*_(1,14)_ = 1.244, *p *=* *0.2835; ***B***, Sex × Day: *F*_(1,14)_ = 0.01586, *p *=* *0.9016; Sex: *F*_(1,14)_ = 5.719e005, *p *=* *0.9941; Day: *F*_(1,14)_ = 0.2869, *p *=* *0.6006 Download Figure 3-1, EPS file.

Next, the Hargreaves test was repeated. In this test, latency to withdraw the paw is used as an index of thermal sensitivity, where increased withdrawal latency is indicative of decreased thermal sensitivity. There were no significant effects of time, EtOH, or sex on withdrawal latency^r^ (Fig. 3*D*). Next, correlations examining the relationship between EtOH intake and withdrawal latency were performed. Previous day EtOH intake was directly correlated with withdrawal latency in males but not females^s^ (Fig. 3*E*). This association was also seen when examining next day EtOH intake, where withdrawal latency predicted subsequent EtOH intake in males but not females^t^ (Fig. 3*F*). The correlation of paw withdrawal threshold and EtOH intake in males as compared with females was statistically significant^s,t^ (Fig. 3*E*,*F*). Together, these findings reveal that increased EtOH intake is bidirectionally associated with decreased thermal sensitivity in a sex-specific manner. Finally, there were no changes in 24-h EtOH intake produced by behavioral testing (Extended Data Fig. 3-1).

### EtOH drinking and associated affective behavior

**Figure 4.**
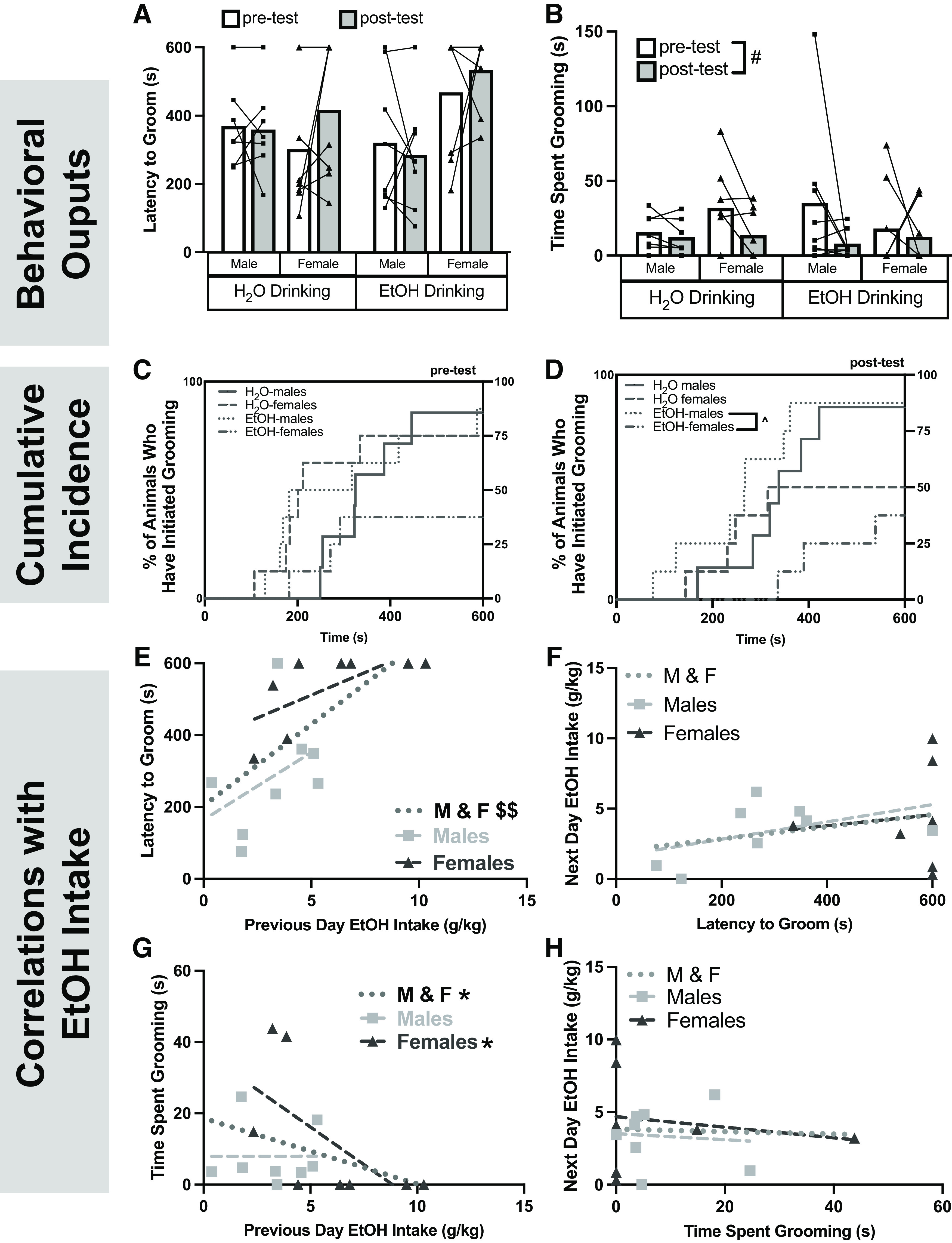
Affective behavior in the splash test following chronic EtOH drinking in males and females. Latency to groom (***A***) and time spent grooming (***B***) were used as measure of negative affect. To account for animals that failed to groom within the 10 min testing period, cumulative incidence curves for the pre-test and post-test are shown in ***C*** and ***D***, respectively. The splash test was repeated and affective behavior was correlated with previous day EtOH intake (***E***, ***G***) or subsequent EtOH intake (***F***, ***H***). Data are presented as individual points. When applicable, bars represent mean. This behavioral test did not affect EtOH intake, as demonstrated in Extended Data Figure 4-1. #*p *<* *0.05 (three-way ANOVA, main effect of time); ^*p *<* *0.05, Mantel–Cox log-rank test followed by Bonferroni *post hoc*, $*p *<* *0.05 (Pearson’s correlation); *p <* *0.05 (Spearman’s correlation). Pearson’s correlations: ***E***, M&F: *r*_(14)_ = 0.6108, *p* = 0.0120; Males: *r*_(6)_ = 0.4120, *p *=* *0.3105; Females: *r*_(6)_ = 0.6172, *p *=* *0.1030; ***F***, M&F: *r*_(13)_ = 0.2592, *p* = 0.3508; Males: *r*_(6)_ = 0.4774, *p *=* *0.2269; Females: *r*_(5)_ = 0.1107, *p *=* *0.3132. Spearman’s correlations: ***G***, M&F: *r*_s(14)_ = −0.5859, *p *=* *0.0193; Males: *r*_s(6)_ = −0.8456, *p *=* *0.0179; Females: *r*_s(6)_ = 0.07,143, *p *=* *0.0179. ***H***, M&F: *r*_s(13)_ = −0.02,951, *p *=* *0.9180; Males: *r*_s(6)_ = 0.09,524, *p *=* *0.8401; Females: *r*_s(5)_ = −0.1782, *p *=* *0.7182.

To examine the effects of chronic EtOH intake on affective behavior, the splash test was repeated after five weeks of voluntary drinking. There was no effect of either EtOH drinking or sex on latency to groom^u^ (Fig. 4*A*) or total time spent grooming^v^ (Fig. 4*B*). However, a main effect of time emerged on total time spent grooming, where all rats (regardless of sex or history of EOH drinking) spent significantly less time grooming on the post-test.

10.1523/ENEURO.0055-23.2023.f4-1 Extended Data Figure 4-1 Splash test testing does not affect EtOH Intake. Two-way ANOVA: Sex × Day: *F*_(1,13)_ = 1.958, *p *=* *0.1852; Sex: *F*_(1,13)_ = 1.883, *p *=* *0.1932; Day: *F*_(1,13)_ = 1.989, *p *=* *0.1819 Download Figure 4-1, EPS file.

To account for animals that failed to groom during the 10-min test period, the cumulative incidence of grooming was plotted using the Kaplan–Meier survival curve followed by the Mantel–Cox log-rank test. There were no differences in cumulative incidence of grooming during the pre-test^w^ (Fig. 4*C*). However, there was a significant effect during the post-test^x^ (Fig. 4*D*), specifically where there was an increased cumulative incidence of grooming in EtOH-drinking males compared with EtOH-drinking females^x^. This suggests sex differences in latency to groom occur only in rats with a history of EtOH drinking (and not in EtOH-naive rats).

To further investigate the association between EtOH intake and affective behavior, individual EtOH intake during the drinking session immediately before and after the splash test were compared with latency to groom and total time spent grooming. Rats that consumed more EtOH before testing took significantly longer to begin grooming^y^ (Fig. 4*E*), an effect that was only seen when sexes were combined. This relationship was unidirectional, as latency to groom did not predict subsequent drinking^z^ (Fig. 4*F*). Total time spent grooming was also associated with EtOH intake before testing such that rats that consumed more EtOH groomed less^aa^ (Fig. 4*G*); however, this effect was primarily driven by females^w^. Additionally, there was no association between total time spent grooming and subsequent EtOH intake^ab^ (Fig. 4*H*) and the splash test did not affect EtOH intake (Extended Data Fig. 4-1). Together, these data suggest that previous day EtOH intake can predict both latency to groom and time spent grooming in the splash test.

**Figure 5.**
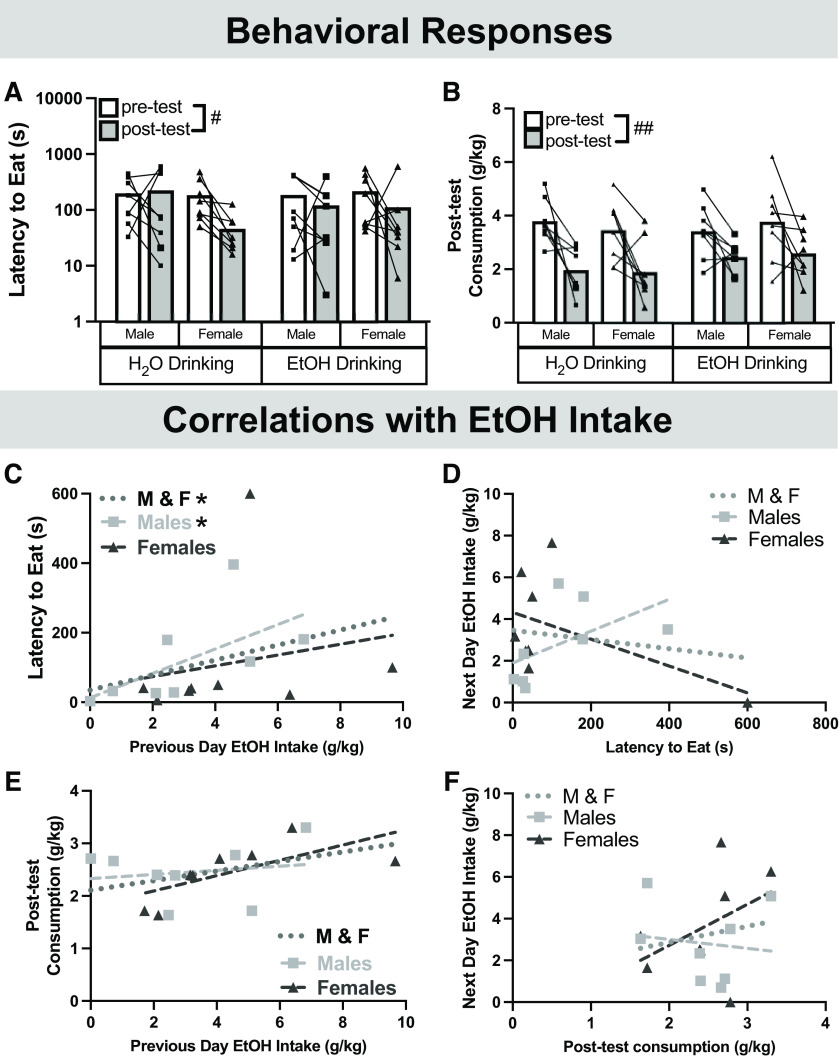
Affective behavior in the novelty suppressed feeding test following chronic EtOH drinking in males and females. Latency to eat (***A***) and post-test consumption (***B***) were used as measured of affective and appetitive behavior. Cumulative incidence of feeding initiation is shown in Extended Data Figure 5-1*A*,*B*. Affective behavior was correlated with previous day EtOH intake (***C***) or subsequent EtOH intake (***D***). Appetitive behavior was not correlated with previous day EtOH intake (***E***) or subsequent EtOH intake (***F***). This test did not affect EtOH intake, as shown in Extended Data Figure 5-1*C*. There were no differences in body weight change, nor did the change correlate with latency to eat or post-test consumption, as shown in Extended Data Figure 5-2. #*p *<* *0.05, ##*p *<* *0.01 (three-way ANOVA, main effect of time); $*p *<* *0.05 (Pearson’s correlation); *p <* *0.05 (Spearman’s correlation). Data are presented as individual points. When applicable, bars represent mean. Spearman’s correlations: ***C***, M&F: *r*_s(14)_ = 0.5647, *p *=* *0.0248; Males: *r*_s(6)_ = 0.7381, *p *=* *0.0458; Females: *r*_s(6)_ = 0.4286, *p *=* *0.2992; ***D***, M&F: *r*_s(14)_ = 0.1912, *p *=* *0.4769; Males: *r*_s(6)_ = 0.6667, *p *=* *0.0831; Females: *r*_s(6)_ = −0.1905, *p *=* *0.6646. Pearson’s correlations: ***E***, M&F: *r*_(14)_ = 0.4202, *p *=* *0.1051; Males: *r*_(6)_ = 0.1630, *p *=* *0.6997; Females: *r*_(6)_ = −0.6833 *p *=* *0.0617; ***F***, M&F: *r*_(14)_ = 0.1875, *p *=* *0.4867; Males: *r*_(6)_ = −0.1263, *p *=* *0.7657; Females: *r*_(6)_ = 0.4296 *p *=* *0.2881.

The NSF test was also repeated to investigate changes in latency to eat and home-cage consumption following EtOH drinking. While there was no effect of sex or EtOH drinking on latency to eat^ac^, there was an effect of time^ac^ where all rats decreased their latency to eat on the second test (Fig. 5*A*). Post-test home-cage consumption was monitored for 10 min following the test. Compared with baseline, all the rats ate significantly less in their home-cage (Fig. 5*B*). There were no differences in cumulative incidence of feeding initiation during baseline or after chronic drinking (Extended Data Fig. 5-1) suggesting that while rats decreased their latency to eat in the NSF, there were no differences between the EtOH and water-drinking groups.

10.1523/ENEURO.0055-23.2023.f5-1 Extended Data Figure 5-1 Cumulative occurrence of behavioral initiation in the NSF is not affected by EtOH drinking, nor is EtOH intake affected by NSF testing. There was no effect of group on cumulative occurrence of feeding initiation in the pre-test (***A***; Mantel–Cox log-rank test; χ^2^ = 0.5295, df = 3, *p *=* *0.9124) or post-test (***B***; Mantel–Cox log-rank test; χ^2^ = 3.717, df = 3, *p *=* *0.2937). Lastly, there was no effect of testing on EtOH intake (**C**; two-way ANOVA; Sex × Day: *F*_(1,13)_ = 0.5462, *p *=* *0.4721; Sex: *F*_(1,14)_ = 1.901, *p *=* *0.3442; Day: *F*_(1,14)_ = 1.901, *p *=* *0.1896). Download Figure 5-1, EPS file.

10.1523/ENEURO.0055-23.2023.f5-2 Extended Data Figure 5-2 Percent change in body weight does not account for effects seen in NSF. There was no difference in body weight changes (***A***; two-way ANOVA; Sex × EtOH: *F*_(1,28)_ = 0.01105, *p *=* *0.9170; Sex: *F*_(1,28)_ = 1.998, *p *=* *0.1685; EtOH: *F*_(1,28)_ = 0.002337, *p *=* *0.9618). Body weight change was not associated with latency to eat (***B***; M&F: *r*_s(14)_ = −0.01471, *p *=* *0.9607; Males: *r*_s(6)_ = −0.2619, *p *=* *0.5364; Females: *r*_s(6)_ = 0.02381, *p *=* *0.9768) or post-test consumption (***C***; M&F: *r*_(14)_ = −0.2642, *p *=* *0.3228; Males: *r*_(6)_ = −0.2771, *p *=* *0.5064; Females: *r*_(6)_ = −0.2744 *p *=* *0.5107). One important consideration is that body weights were not measured immediately before food deprivation (and instead were taken 1 week prior to the test). However, body weights were taken on the day of the NSF test (i.e., following 24 h of food deprivation). Percent body weight was calculated as [100 + 100 × (body weight_NSF day_ – body weight_1 week prior_)/body weight_1 week prior_]. Download Figure 5-2, EPS file.

Associations between NSF behavioral measures and EtOH drinking were performed to examine potential individual effects of EtOH drinking on behavior. Rats that consumed more EtOH before the NSF test had increased latencies to eat^ae^, and this effect was driven primarily by males (Fig. 5*C*). There were no significant correlations between latency to eat and subsequent EtOH intake^af^ (Fig. 5*D*). Post-test home-cage consumption did not correlate with previous day^ag^ (Fig. 5*E*) or subsequent EtOH intake^ah^ (Fig. 5*F*). Similar to the splash test, there was no effect of testing on EtOH intake in either sex (Extended Data Fig. 5-1). Additionally, there was no effect of EtOH or sex on changes in body weight, nor did body weight change correlate with NSF behavioral measures (Extended Data Fig. 5-2). Together, these correlations suggest that EtOH intake is correlated with subsequent latency to eat on the NSF test.

### EtOH drinking and medial prefrontal cortex activity

Following the NSF test, rats were allowed one final drinking session before brains were collected and the medial prefrontal cortex was stained for RFP (to identify CRF1+ cells) and cFos (a marker of neuronal activity). The medial prefrontal cortex was divided into prelimbic (PL) and infralimbic (IL) cortices, based on the Paxinos and Watson atlas (Paxinos and Watson, 2007).

**Figure 6.**
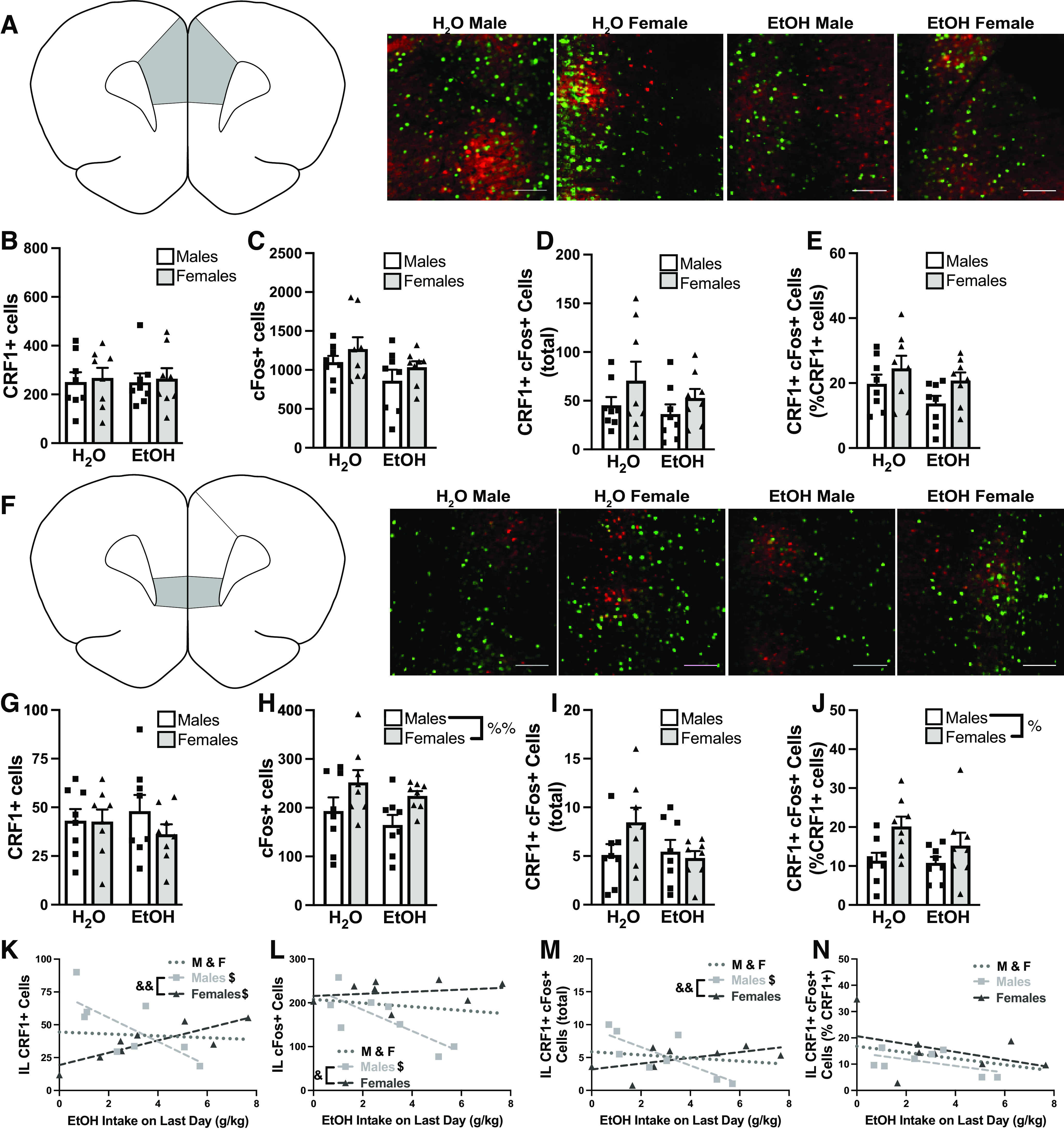
Prefrontal correlates of EtOH drinking in males and females. Slices containing both the prelimbic cortex (PL) and infralimbic cortex (IL) were stained for RFP (as a measure of CRF1+ neurons, shown in red) and cFos (as a measure of cellular activity, shown in green). PL representative images (***A***) showing CRF1+ (***B***), cFos+ (***C***), and total double labeled cells (***D***). Double labeled cells were also expressed as % total CRF1+ cells (***E***). Cell counts in the PL did not correlate with EtOH intake, as shown in Extended Data Figure 6-1. IL representative images (***F***) showing CRF1+ (***G***), cFos+ (***H***) and total double labeled cells (***I***). Double labeled cells were also expressed as % total CRF1+ cells (***J***). We also correlated EtOH intake on the last day with IL labeling of CRF1+ cells (***K***), cFos+ cells (***L***), total double labeled cells (***M***) and double labeled cells as %CRF1+ (***N***). Data are presented as individual points. When applicable, bars represent mean + SEM. Scale bars= 100 μm; %% *p *<* *0.01 (two-way ANOVA; main effect of sex); $*p *<* *0.05 (Pearson’s correlation); &&*p *<* *0.01 (Fisher’s *r* to *z* transformation). Pearson’s correlations: ***K***, M&F: *r*_(14)_ = −0.07,998, *p *=* *0.7684; Males: *r*_(6)_ = −0.7277, *p *=* *0.0407; Females: *r*_(6)_ = 0.8307, *p *=* *0.0106; ***L***, M&F: *r*_(14)_ = −0.1695, *p *=* *0.5403; Males: *r*_(6)_ = −0.7775, *p *=* *0.0231; Females: *r*_(6)_ = 0.2192, *p *=* *0.6022; ***M***, M&F: *r*_(14)_ = −0.1909, *p *=* *0.4787; Males: *r*_(6)_ = −0.7752, *p *=* *0.0238; Females: *r*_(6)_ = 0.5508, *p *=* *0.1571; ***N***, M&F: *r*_(14)_ = −0.3485, *p *=* *0.1858; Males: *r*_(6)_ = −0.5444, *p *=* *0.1630; Females: *r*_(6)_ = −0.4090, *p *=* *0.3144.

When investigating PL (Fig. 6*A*), there were no differences between EtOH-drinking and water-drinking groups when examining CRF1+ cells^ai^ (Fig. 6*B*), cFos+ cells^aj^ (Fig. 6*C*), total double-labeled cells^ak^ (Fig. 6*D*), or double-labeled cells as a percentage of total CRF1+ cells^al^ (Fig. 6*E*). Additionally, final session EtOH intake was not associated with any of these cell counts (Extended Data Fig. 6-1). In the IL (Fig. 6*F*), there was no effect of sex or EtOH on CRF1+ cells^am^ (Fig. 6*G*). However, females had increased cFos+ cells^an^ (Fig. 6*H*). There were no significant differences in total double-labeled cells^ao^ (Fig. 6*I*), but the increased expression in females seen in Figure 6*H* persisted when double-labeled cells were expressed as %CRF1+ cells^ap^ (Fig. 6*J*). The number of CRF1+ cells was directly correlated with EtOH intake in females^aq^ but was inversely correlated with EtOH intake in males^aq^ (Fig. 6*K*). Additionally, the number of cFos+ cells was inversely correlated with EtOH intake in males only^ar^ (Fig. 6*L*). Consistent with the correlations seen thus far, total double-labeled cells was inversely correlated with EtOH intake in males only^as^ (Fig. 6*M*); however, this correlation disappeared when double labeled cells were normalized to %CRF1+ cells^at^ (Fig. 6*N*). Taken together, these findings highlight differential effects of EtOH drinking on medial prefrontal cortex subdivisions, and suggest sex-dependent and dose-dependent effects of EtOH on IL CRF1+ and cFos+ cells.

10.1523/ENEURO.0055-23.2023.f6-1 Extended Data Figure 6-1 Correlations between EtOH intake and activity in the prelimbic cortex. EtOH intake did not correlate with CRF1+ expression (***A***), cFos+ cells (***B***), total double-labeled cells (***C***) or double-labeled cells when expressed as %CRF1+ cells (***D***). Spearman’s correlations: ***A***, M&F: *r*_s(14)_ = −0.1735, *p *=* *0.5193; Males: *r*_s(6)_ = −0.5476, *p *=* *0.1710; Females: *r*_s(6)_ = 0.09524, *p *=* *0.8401. Pearson’s correlations: ***B***, M&F: *r*_(14)_ = −0.1997, *p *=* *0.4582; Males: *r*_(6)_ = −0.25643, *p *=* *0.5271; Females: *r*_(6)_ = −0.3325, *p *=* *0.4210); ***C***, M&F: *r*_(14)_ = −0.07430, *p *=* *0.7842; Males: *r*_(6)_ = −0.2966, *p *=* *0.4757; Females: *r*_(6)_ = −0.02222, *p *=* *0.9583; ***D***, M&F: *r*_(14)_ = −0.01427, *p *=* *0.9582; Males: *r*_(6)_ = −0.1604, *p *=* *0.7043; Females: *r*_(6)_ = −0.09422, *p *=* *0.82. Download Figure 6-1, EPS file.

## Discussion

The current study examined the dynamic relationship between affective state, pain sensitivity, and alcohol intake in male and female CRF1:cre:tdTomato rats. While female rats displayed increased basal sensitivity to mechanical stimuli, there were no other sex differences in baseline behavior. Females consumed significantly more EtOH during the first week of intermittent access, but overall EtOH intake was not significantly different between males and females. EtOH drinking decreased mechanical sensitivity in both male and female rats, but mechanical sensitivity did not correlate with previous or subsequent EtOH intake. Conversely, there were no group effects of EtOH on thermal sensitivity; but in males, decreased thermal sensitivity was associated with increased EtOH intake before and after the Hargreaves test. There were no group effects of EtOH on behaviors in the splash test or NSF test; however, affective behavior was directly correlated with EtOH intake before testing, but not with subsequent EtOH intake. Additionally, all groups displayed increased mechanical sensitivity, decreased time spent grooming in the splash test, and decreased home-cage consumption during the NSF test when compared with their baseline levels. Finally, we investigated potential effects of EtOH intake on the activity of CRF1-containing neurons in the prelimbic and infralimbic prefrontal cortex. While there were no significant group effects of EtOH drinking on overall or CRF1-specific activity, there were significant sex-specific associations between EtOH intake and activity in the infralimbic region. These findings provide new insight into individual differences in EtOH intake and its association with affective state, nociception, and neuronal activation of CRF1-containing neurons in key regions of the prefrontal cortex.

One important goal of this study was to examine affective and nociceptive behavior and EtOH drinking in both sexes of CRF1:cre:tdTomato rats. There was no effect of sex on basal affective behavior with the exception of mechanical sensitivity, where female rats displayed increase mechanical sensitivity. One previous study examined pain sensitivity in male and female CRF1:cre:tdTomato rats; however, data were not reported by sex (Weera et al., 2022). Compared with that study (as well as other work in Wistar rats; Ririe and Eisenach, 2006; Edwards et al., 2012; Geng et al., 2016; Pahng et al., 2017), our rats had significantly lower withdrawal thresholds. This discrepancy could be because of stress associated with social isolation, as others have found social isolation increases mechanical sensitivity (Horiguchi et al., 2013; Nishinaka et al., 2015). Alternatively, the lower thresholds may be because of including differences in housing environment, breeding/transport differences, or equipment/testing environment differences including social transfer of stress (M.J. Smith et al., 2016, 2017, 2021; Sterley et al., 2018). Interestingly, previous work in our lab using rats had more typical paw withdrawal threshold values (Quadir et al., 2022).

Future studies will investigate these discrepancies further and in greater depth. Studies involving Wistar rats (the background strain for the CRF1:cre:tdTomato rats) also found no sex differences in baseline affective behavior in the splash test (Keledjian et al., 2020; Abelaira et al., 2022). Sex differences in the NSF test in Wistar rats are less clear: one study found females exhibited increased affective behavior (De Oliveira Sergio et al., 2021); however, other studies report no sex differences (Francis-Oliveira et al., 2013; Cabbia et al., 2018; Enayati et al., 2020). Following baseline behavioral testing, rats were allowed to voluntarily drink EtOH under an intermittent access paradigm. In this study, male and female CRF1:cre:tdTomato rats drank 2–4 g/kg EtOH with an approximate 15–30% EtOH preference, consistent with studies in Wistar rats (Adermark et al., 2011; Cippitelli et al., 2012; Momeni and Roman, 2014; Kimbrough et al., 2017; Albrechet-Souza et al., 2020; Scott et al., 2020; Moench and Logrip, 2021). While there were no sex differences in EtOH intake throughout the experiment, females drank significantly more EtOH during the first week. Studies comparing male and female EtOH intake under intermittent access conditions in Wistar rats are limited and inconclusive: some researchers found that females drank more EtOH whereas others found no sex differences (Priddy et al., 2017; Albrechet-Souza et al., 2020). Further studies will examine EtOH drinking in other contexts, such as binge-drinking or drinking following abstinence from EtOH. The studies presented here expand our knowledge of baseline behavior and EtOH intake in male and female CRF1:cre:tdTomato rats and provide a key foundation for future studies.

Although the behavioral tests used in these studies examine different aspects of affective and nociceptive behavior, one common theme emerged in the postdrinking tests: these behaviors increased in both EtOH drinking animals as well as EtOH-naive animals. Specifically, we found increases in the Von Frey test (decreased withdrawal thresholds, suggesting increased mechanical sensitivity), splash test (decreased time spent grooming, suggestive of increased negative affect), and NSF test (decreased post-test consumption, suggestive of decreased appetite). Our study aimed to investigate the directionality of the relationship between negative affect, nociception, and EtOH drinking; therefore, having baseline measures was crucial. However, affective tests are rarely repeated in the same subjects; thus, there are limited findings regarding the effects of time and re-testing on behavior. Our lab previously demonstrated that male rats, but not female rats, spent significantly less time grooming on the second splash test (Quadir et al., 2022).

Additionally, test conditions were not changed between pre-test and post-test. Although this was intentionally designed based on findings that demonstrate three weeks is sufficient to wait between testing (Adamec and Shallow, 2000; Adamec et al., 2005; Blasio et al., 2014; Goes et al., 2015), we cannot rule out the possibility that associations between negative affect and EtOH intake may actually reflect memory impairments and EtOH intake. Indeed, repeated studies show that EtOH exposure can impair memory retrieval (Land and Spear, 2004; Houlé et al., 2017). Another important caveat is that these tests are designed to present a novel anxiogenic environment to the animal being tested. However, with repeated testing, the environment loses its novelty. Thus, the main effect of time decreasing in latency to eat in the NSF may simply reflect that the test environment was no longer novel. Interestingly, this did not occur when investigating the latency to groom in the splash test, suggesting the possibility of test-specific memory. One explanation is the food deprivation stress along with the bright lighting (150 lux) in the NSF resulted in stronger memory formation (Ye et al., 2018; Stelly et al., 2020). The splash test, on the other hand, was conducted without food restriction and in a darker environment; thus, the stress of the splash test may not have been significant enough to induce a stronger context-induced memory. Similar to this, the novelty of the Hargreaves and Von Frey apparatuses may have resulted in stress-induced analgesia, a phenomenon seen repeatedly in Wistar rats (Vendruscolo et al., 2004, 2006) as well as in mice (Kambur et al., 2008; Kurrikoff et al., 2008), which may have contributed to the decreased pain thresholds seen at Von Frey re-testing.

Many studies have reported increased negative affect and nociception following chronic EtOH drinking (Overstreet et al., 2005; Van Skike et al., 2015; Gong et al., 2017; Quadir et al., 2021; Xu et al., 2021). However, there were no group effects of EtOH on thermal sensitivity or affective behavior in the studies presented here. One potential explanation is that rats in the present study only consumed EtOH for three to four weeks before behavioral testing and thus did not consume enough EtOH to produce behavioral deficits. Studies in Wistar rats report escalated intake after ∼20 EtOH drinking sessions (Carnicella et al., 2009; George et al., 2012; Kimbrough et al., 2017), whereas rats in this study only drank for 16 sessions. Others have found that various aspects of EtOH withdrawal (including increased negative affect behavior, seizures, and EtOH intake during withdrawal) increase with repeated EtOH withdrawal cycles (Becker, 1996; Holter et al., 1998; Rimondini et al., 2003; Overstreet et al., 2004; Lopez and Becker, 2005; Z. Zhang et al., 2007). Another possibility is that the environment was too anxiogenic, creating a ceiling effect on negative affect. Recent studies have developed methods to test affective behavior in the home-cage (Neira et al., 2022) to remove this potential confound and will be considered in future work.

Despite the lack of group effects of EtOH, there were significant individual correlations between behavioral readouts and EtOH intake, thus highlighting the importance of examining individual differences in the context of EtOH-related behaviors. More specifically, this study found previous EtOH intake correlated with thermal sensitivity as well as alterations in affective behavior. There was an inverse relationship between EtOH intake and thermal sensitivity, where males that consumed more EtOH were less sensitive to thermal stimuli and males with increased thermal sensitivity consumed less EtOH on the next day. These findings are in accordance with a previous study reporting that EtOH withdrawal increases nociceptive threshold (Schunck et al., 2015). However, it is important to note that other studies have demonstrated EtOH-induced decreases in pain threshold (Avegno et al., 2018; Kononoff et al., 2018; Walcott et al., 2018; S. Kang et al., 2019b; You et al., 2020). One potential explanation is that EtOH-induced peripheral neuropathy blunted the ability of the rats to feel the thermal stimulus. This would be consistent with the group effect of EtOH on mechanical sensitivity, where the EtOH group had increased paw withdrawal thresholds, corresponding to decreased mechanical sensitivity. An alternative possibility is that CRF1:cre:tdTomato rats may be resistant to the analgesic properties of EtOH; however, additional studies are required to examine this in more detail. One final possibility is that male EtOH rats are more sensitive to stress-induced analgesia. Studies have found brief exposures to forced swim and predator odor decreased thermal nociception in both male and female Wistar rats (Vendruscolo et al., 2004, 2006), but whether chronic low doses of EtOH drinking is a sufficient stressor to induce hyperalgesia remains unclear.

In addition to associations with thermal sensitivity, previous day EtOH intake was also associated with negative affect in the splash test and NSF test. These associations were specific to the EtOH group as they are lost when water-drinking animals are included in the analysis (data not shown), likely because of the loss of dynamic range when EtOH intake is zero for all subjects. Collectively, these results suggest that while basal negative affect is variable and likely determined by a number of factors, chronic drinking does contribute to EtOH-affective state interactions. These findings are consistent with previous studies that demonstrate increased fear-potentiated startle in various genetic lines of alcohol preferring rats (McKinzie et al., 2000) and mice (Barrenha and Chester, 2007). While all of these correlations held true when both sexes were analyzed together, analyzing the sexes separately revealed that the EtOH intake associations with negative affect in the NSF were primarily driven by males whereas EtOH intake associations with negative affect in the splash test were primarily driven by females. These findings suggest test-specific contributions of sex in EtOH-induced alterations in affective behavior. Interestingly, these behaviors were unable to predict subsequent EtOH intake in either sex. Other studies have mixed findings regarding associations of affective behavior and subsequent drinking with significant correlations found in some studies (Spanagel et al., 1995; Hayton et al., 2012; Pelloux et al., 2015) but not others (Henniger et al., 2002; Da Silva et al., 2004; Correia et al., 2009; McNamara and Ito, 2021).

The effects of alcohol on affective and nociceptive behaviors are known to be mediated by key brain regions including the medial prefrontal cortex. Many studies fail to differentiate the prelimbic (PL) and infralimbic (IL) subdivisions of this region, but it is clear that these regions often play opposing roles in mediating behavior. For example, dopamine blockade in the PL reduces place conditioning without affecting cue conditioning but dopamine blockade in the IL reduces cue conditioning without affecting place conditioning (Hayen et al., 2014). While both PL and IL are active during cue-induced reinstatement of EtOH seeking, only ablation of activity-dependent IL neurons inhibited EtOH seeking (Pfarr et al., 2015). Another study found that EtOH drinking during adolescence increased Arc immunoreactivity in PL (but not in IL), whereas EtOH drinking during adulthood increased Arc immunoreactivity in IL (but not PL; Lawson et al., 2022). While the lack of effect in PL is consistent with the present findings, the effects in IL were divergent in that our study found decreased activity in the IL whereas their study found increased IL activity. In addition to species differences (mice vs rats), the timing of tissue collection may explain the differences seen. In the referenced study, brains were collected 25 d after the last drinking session, but in the studies presented here, brains were collected 24 h after the last drinking session. It is possible that increased length of abstinence results in compensatory upregulation of neuronal activity. Indeed, many studies have demonstrated opposing effects during short-term abstinence (∼24 h) and long-term abstinence (>7 d; Cannady et al., 2020; R.J. Smith et al., 2020).

PL and IL also exert opposing effects on stress reactivity, especially in the context of EtOH. One important study found that the CRF1 antagonist CP154,526 injected directly into the IL could inhibit stress-induced reinstatement of EtOH seeking, an effect that did not occur if the drug was injected directly into the PL (Flores-Ramirez et al., 2022). These studies highlight the necessity of investigating PL and IL as separate regions, rather than combining them into one, particularly in the context of EtOH and behavior. In the studies presented here, there were no group differences or individual differences in the effects of chronic EtOH drinking on neuronal activation (cFos+ cells) or activation of CRF1+ cells (double labeled cells) in the PL. However, individual differences emerged when examining the association between EtOH intake and cFos+ cells as well as EtOH intake and activated CRF1+ cells in the IL. Not only were these associations region-specific, but also sex-dependent as the correlations only occurred in male rats. There are limited studies investigating the effects of EtOH drinking in CRF1+ cells in the PL and IL subdivisions of the PFC. One study investigated the effects of EtOH self-administration and extinction on *Crhr1* mRNA levels in the IL of male rats and found that three weeks of self-administration followed by 10d of extinction decreased *Crhr1* transcript levels in the IL (Flores-Ramirez et al., 2022). This is in line with our findings that demonstrate EtOH drinking is associated with decreased activity of CRF1+ cells in the IL. However, future studies manipulating the activity of these cells will need to be done to confirm a direct role of CRF1+ cells in mediating EtOH intake. Researchers have also reported that CRF1 antagonists injected directly into the PFC (not separating PL and IL) blunts EtOH intake (Robinson et al., 2019). One important consideration is that most prior studies examined changes in CRF1 transcript levels or CRF1 activity via antagonists; however, our studies examined the effects of EtOH on cells that contain CRF1 and thus cannot be compared directly. One important study found that EtOH vapor withdrawal decreases excitability and excitatory neurotransmission in CRF1+ neurons in the prefrontal cortex (Patel et al., 2022); however, this study was only performed in male mice and did not separate PL from IL. One final limitation from these findings is that cFos expression peaks at 30–90 min following a stimulus and returns to baseline levels within 12 h (Barros et al., 2015), complicating the ability to examine associations between neuronal activity and affective/nociceptive behaviors. Future studies will examine ΔFosB expression, as it is marker of longer-term neuronal activity. Collectively, these studies highlight the relevance of including both sexes and subregions while examining the effects of chronic EtOH drinking. One final limitation is this work only focused on one region of the brain, the medial PFC. There are many other brain regions that are implicated in EtOH drinking, affective behavior, and nociception that are also rich with CRF and CRF1. These regions include the central amygdala, bed nucleus of the stria terminalis, and periaqueductal gray (Ji et al., 2007; Litvin et al., 2007; Francesconi et al., 2009; Miguel and Nunes-de-Souza, 2011; Gozzi et al., 2013; Ide et al., 2013; Tran et al., 2014; Faria et al., 2016; Natividad et al., 2017; Avegno et al., 2018; Agoglia et al., 2022). These areas are outside the scope of this project but will be investigated in the future.

Together, our results illustrate the complex interplay between affective state, EtOH drinking, and the role of prefrontal cortex CRF1+ neurons in mediating these behaviors. Moreover, these data highlight the importance of examining individual differences in addition to group averages when investigating AUD-related behaviors and affective states. There are currently only four FDA-approved medications to treat AUD, and persistent issues with efficacy and compliance complicate effective treatment. Clinical trials have demonstrated differential efficacies of medications when stratified by factors such as sex, age of AUD onset, severity of AUD, and impulsivity levels (Zorrilla et al., 2013; Leggio et al., 2020). However, co-morbidity of AUD and affective disorders remains an underexamined issue. Thus, additional studies that consider individual differences and sex in affective behavior and population-specific neurobiological changes in the context of AUD are warranted.

## Synthesis

Reviewing Editor: Leandro Vendruscolo, National Institute on Drug Abuse Decisions are customarily a result of the Reviewing Editor and the peer reviewers coming together and discussing their recommendations until a consensus is reached. When revisions are invited, a fact-based synthesis statement explaining their decision and outlining what is needed to prepare a revision will be listed below. The following reviewer(s) agreed to reveal their identity: Candice Contet.

N/A

## Author Response

Response to Reviewers

We thank the reviewers for the time and attention to this manuscript. We believe the points raised were important and have done our best to address all of the changes suggested by the reviewers. We feel the work has been much improved by their suggestions. Below, please find each concern raised by the reviewer and our response. Changes in the manuscript and supplemental material are indicated in blue text throughout.

Reviewer 1

1. Given the emphasis of the study on nociception and affect, the focus on prelimbic and infralimbic cortex for histological analysis is weakly justified. The study would be strengthened by including additional CRF1-rich regions where CRF1 signaling has been implicated in modulating alcohol drinking, affective behaviors, and/or pain thresholds. Furthermore, it might be fruitful to also analyze the correlation between CRF1+ and cFos+ counts and nociceptive/affective readouts. For instance, based on ref 50/52, lower von Frey nociceptive thresholds of females at baseline may correlate with higher activity of central amygdala CRF1+ neurons.

We performed additional correlational analyses between the IHC components and the behaviors mentioned and found no significant correlations between CRF1+ cells, double labeled cells, or %CRF1+ labeled cells in either sub-region and affective/nociceptive readouts. However, thermal sensitivity correlated with cFos+ cells in the prelimbic cortex in drinking females, and mechanical sensitivity correlated directly with cFos+ cell counts in the infralimbic cortex of drinking males. We hesitate to add these findings to the manuscript due to the timing of behavioral testing and tissue collection: NSF was performed 3d before tissue collection, splash test was performed 8d before tissue collection, Hargreaves was performed 13d before tissue collection, and Von Frey was performed 18d before tissue collection. Because cFos is considered an immediate early gene with peak expression 30-90min following an event and levels drop to control levels within 12h (PMID: 25814929), this timing creates a significant complication for comparing cFos expression to behavioral testing that occurred 3-18d before tissue collection. We have added text to the discussion acknowledging this as a limitation.

Additionally, we recognize that the manuscript may be strengthened by adding immunohistochemical analysis of more CRF rich-regions that have also been implicated in alcohol, affect, and pain. However, the focus of this work was on the less explored CRF-rich regions prelimbic and infralimbic subregions of the PFC. Additional areas will be the focus of future studies. We have added additional justification for studying the PFC in the introduction and have also included a paragraph in the discussion regarding the more traditional regions.

2. The unique finding of this study, which was afforded by the use of CRF1 reporter rats, relates to the sex-specific correlation between alcohol intake and activation of infralimbic CFR1-expressing cells. Testing the behavioral significance of this observation (by manipulating CRF1 signaling or CRF1+ cell activity in the infralimbic cortex) would greatly enhance the impact of the study. At the very least, published studies that have performed such manipulations in the context of alcohol self-administration should be referenced and discussed (cf. PMID 35341789).

We agree with the reviewer that manipulation of CRF1+ neurons in the infralimbic cortex would be of significant interest and plan to explore these manipulations in greater detail in future/ongoing studies. We have added discussion of the paper referenced (as well as another) into the discussion. We have also added text to the discussion regarding future studies that will test the behavioral significance of the observation.

3. The manuscript would benefit from clearly laying out in the introduction (and abstract) the hypotheses that this study was designed to test and the gaps of knowledge it sought to fill given that sex differences in nociception, affect, and alcohol drinking had already been examined by previous rat studies. It would also be helpful to explain from the outset the rationale for conducting this study in CRF1 reporter rats and why a duration of 3-4 weeks of intermittent alcohol drinking was selected prior to repeating nociceptive/affective testing (while a longer duration was known to be needed to produce intake escalation).

Specific hypotheses have now been added to both the abstract and introduction. In the introduction, we have added rationale for the shorter duration drinking experiments as well as why the study was done in CRF1 reporter rats. By performing these studies in CRF1-cre transgenic rats, we confirm the sex differences and behaviors seen in previous studies but in this transgenic line for the first time. Additionally, we identify the PFC as a target for sex-specific differences in CRF1 activity and lay the groundwork for manipulating the CRF1-containing neurons in future studies.

4. The interpretation of the affective measures seems arbitrary. Why would grooming latency reflect anxiety-like behavior but grooming duration would reflect depressive-like behavior? Are these statements related to known effects of anxiolytic or antidepressant drugs on these measures? The NSF latency has been interpreted as an index of anxiety-like behavior in some studies (conflict-based test) and depressive-like behavior in other studies (based on the effect of chronic antidepressant treatment). In the end, those labels are not particularly helpful for data interpretation, unless multiple assays tapping into the same domain yield convergent findings. In the present study, it seems preferable to use objective descriptions of the behavioral responses measured (grooming propensity and refeeding hesitancy).

We have removed the mention of anxiety- and depressive-like behavior from the results section and replaced it with objective descriptions. Additionally, there are mixed interpretations of if these tests measure anxiety- or depressive-like behavior (if not both). Thus, we decided to remove these terms and instead use affective behavior as a collective term throughout the manuscript.

5. As acknowledged in the Discussion, it is possible that repeating the same nociceptive and affective tests after a 3-4 week break masked the effects of alcohol drinking. The effect of repetition/time is interpreted as increased mechanical sensitivity and negative affect, but other parameters confounding data interpretation should be mentioned/discussed. For instance, the hesitancy to eat in the NSF test stems from the novelty of the context in which food is presented. If the same arena was used in both iterations of the NSF test, the context is no longer novel at re-test, which could explain shorter latencies despite a possibly similar affective state. Likewise, the significant correlation between NSF latencies and ethanol intake may be related to an impaired memory of the testing context rather than altered anxiety levels. The same effect of context novelty may also apply to the splash test. For nociceptive tests, stress-induced analgesia may have influenced thresholds to a larger extent at baseline than at re-test. For NSF, differential body weight loss during food deprivation could also explain differential appetite/food consumption.

Thank you for raising these important points.. We have added text to the discussion considering the limitations and alternative possibilities of repeat testing. Additionally, the reviewer brings up a great point about the potential for weight loss during food deprivation to influence appetite. One confounding problem is that body weights were not measured immediately before food deprivation (and instead were taken 1 week prior to the test). However, body weights were taken on the day of the NSF test (ie following 24h of food deprivation). Using these measurements, we examined body weight loss in all 4 groups (shown below) and found no effect of neither sex nor EtOH intake (Sex: F(1,28)=0.002, p=0.96; EtOH: F(1,28)=2.0, p=0.17; Sex x EtOH: F(1,28)=0.011, p=0.92). Additionally, body weight loss did not correlate with latency to eat or home-cage food consumption during NSF. These graphs have now been added to Supplementary Figure 5-2.

6. With the exception of baseline mechanical nociception, the only significant effects reported are correlations among alcohol-drinking rats. However, there is no discussion of whether these correlations hold if the water drinkers are included. This could help interpret the correlations as either a consequence of alcohol exposure (in which case the correlation should be preserved when including water drinkers as their data should be most similar to low-alcohol drinkers) or as a predictor of alcohol drinking propensity (in which case correlations may collapse when including water drinkers since they weren't given a chance to consume alcohol).

We considered the possibility of adding the water drinkers to the correlations, with their EtOH intake being zero. However, correlations represent data that varies in two variables. Thus, by adding the water animals with the intake being 0, we only add samples to one factor (behavioral output) while keeping the other (EtOH intake) constant, artificially deflating the correlations we aim to investigate. We have included the graphs below, with water drinkers shown in navy and you can see that the correlations effectively disappear. However, we did add text to the discussion regarding the relationship between EtOH intake and behavior as more of a predictor of drinking propensity.

7. The von Frey paw withdrawal thresholds reported here are almost 10-fold lower than those reported in ref 50/52 for the same line of rats. Please discuss what could explain this difference.

There could be many reasons for discrepancies in withdrawal thresholds. We agree that it is important to consider our findings in the context of previous work and have added text in the discussion addressing potential reasons for this difference (social isolation).

8. Some methodological details need to be added:

a. In section 2.2, specify the number of sprays and/or sprayed volume, and the area of the rat body that was targeted by the spray(s).

The number of sprays (1), volume (100 μL), and area of the rat body targeted (dorsal coat of the torso) has been added to the methods.

b. In section 2.6, specify the circadian timepoint at which alcohol bottles were introduced/removed. This has been added to the text (ZT 3).

c. Specify how many hours after alcohol access each post-consumption nociceptive and affective test was conducted, and how long after alcohol access was resumed. This has been added to 2.7.

d. Explain why the order of tests was different at baseline vs. post-consumption. This has been added into the text, in section 2.8.

9. Other fixes:

a. In the Introduction, second paragraph, second sentence, replace “are mediated by” by “involve” (CRF is unlikely to be the only mechanism). Thank you for this suggestion, this change has been made.

b. In the Discussion, third paragraph, equating increased mechanical sensitivity to negative affect is a shortcut. Use another term to encapsulate all phenotypes. Throughout the text, we have reworded sentences to better encapsulate these phenotypes, using “affective and nociceptive behaviors” instead.

Likewise, in Results section 3.3, last sentence, replace “any of the affective behaviors” with “any of the nociceptive or affective behaviors”. This has been changed.

c. In Figure 5A, use a linear rather than logarithmic Y-axis to facilitate comparisons between graphs. Using a linear axis on this graph would be inappropriate, as the data do not follow a normal distribution and thus cannot be compared as is with a 3-way ANOVA. However, when data is transformed logarithmically, the data follows a normal distribution and parametric testing can be performed. However, to facilitate comparisons between baseline and post-drinking tests, we have added a log scale graph of baseline NSF latency to Supplemental Figure 1-2.

d. Consolidate refs 50 and 52. The adjustments have been made.

Reviewer 2:

The present study evaluated various behaviors in a transgenic strain of CRF-cre rats using tests of affective behaviors and an intermittent access 2-bottle choice drinking procedure. While it is important to evaluate and characterize behavior in new transgenic strains of animals, there are major problems with this manuscript. There is too much emphasis on the interaction between AUD and affective behavior in this manuscript given that a model of AUD was not used. In the discussion, the authors acknowledged and discussed the short duration that the rats were allowed to drink and the impact that may have had on their results. This is appreciated but the introduction should also reflect that this is not a study of AUD. Additionally, the conclusions of this manuscript on the impact of alcohol drinking on affective behavior and prelimbic and infralimbic activity are an over reach. Additional comments are listed below.

We appreciate this critical feedback. We have removed the term “AUD” from the abstract, and have edited the introduction to instead place emphasis on the interactions between alcohol use and affective behavior (rather than AUD and behavior). We have also added references to publications that found effects of short duration EtOH drinking on affective and nociceptive behaviors to better contextualize the work contained in the present study.

Comments:

The abstract is a bit vague, the method of alcohol drinking should be stated as intermittent 2BC.

This has been adjusted.

In the sentence “EtOH drinking decreased mechanical sensitivity, but no other group effects were observed” - group effects of what? Affective behavior? Please state this.

This has been adjusted and additional text clarifying group effects has been added.

When the statement “group effects,” is used it is not clear if they are talking about alcohol-drinking vs non alcohol drinking rats or male vs. female rats. Please clarify.

This has been adjusted for clarity throughout the abstract and revised manuscript

Regarding the splash test, can the authors include citations of papers that explain why these two grooming-related measures are reflective of anxiety or depressive-like behavior. Same for the NSF test. The original paper by Simms et al. (2008) characterizing intermittent 2BC should be cited as well.

We agree that there are many definitions of anxiety- and depressive-like behavior and that lack of cariy in these terms can confound interpretation and communication of our findings. Thus, we have removed all use of these terms and instead replaced them with the more objective term “affective behavior” throughout the manuscript. At the reviewer's suggestion, we also added citations that use these tests in these ways. The Simms citation has now been added.

Figure 1A. The experimental timeline figure can be improved upon. It would be easier to understand if the authors included the duration of time for all of the components of the experiment, i.e., how long the rats were single-housed and the number of days the baseline and post-drinking behavioral tests conducted. The figure also makes it seem like alcohol drinking only occurred for 3 weeks.

Thank you for this suggestion, we have modified the timeline (shown below for convenience) with hopes of better communicating the duration of alcohol drinking as well as the duration times for all components.

Figures 2B, 3A-C. The baseline and post-test paw withdrawal threshold values are dramatically lower than what is reported in the literature for rats of a comparable age (Ririe & Eisenach 2006, Edwards et al. 2012) This makes it difficult to interpret the results as meaningful on their own or in relation to alcohol intake. The authors should address this major discrepancy.

The reviewer is correct that the paw withdrawal latencies measured here are much lower than previously reported. This could be due to many potential reasons including differences in housing environment, vendor/transport differences, or equipment/testing environment differences. We agree that it is important to consider our findings in the context of previous work and We have now added text in the discussion addressing potential reasons for this discrepancy (social isolation or social transfer of stress).

It is evident in Figures 4A and 4B that grooming behavior was not affected by sex or history of alcohol drinking, therefore it is unclear what Figures 4C and 4D add to this paper.

One limitation of the data presented in figures 4A-B is that some animals never initiated grooming during the 10 min period. As a result, they were assigned latencies of 600s and this assumes that the animals would have groomed immediately if we waited any longer. In reality, by assigning this artificial value, we are reducing the chances of seeing any effect by creating an artificial ceiling. The data presented in 4C and 4D bypass this limitation by instead looking at what proportion of animals have initiated grooming at a given timepoint, making the grooming a discrete rather than continuous variable.
